# Active dendrites regulate the spatiotemporal spread of signaling microdomains

**DOI:** 10.1371/journal.pcbi.1006485

**Published:** 2018-11-01

**Authors:** Reshma Basak, Rishikesh Narayanan

**Affiliations:** Cellular Neurophysiology Laboratory, Molecular Biophysics Unit, Indian Institute of Science, Bangalore, India; Instytut Biologii Doswiadczalnej im M Nenckiego Polskiej Akademii Nauk, POLAND

## Abstract

Microdomains that emerge from spatially constricted spread of biochemical signaling components play a central role in several neuronal computations. Although dendrites, endowed with several voltage-gated ion channels, form a prominent structural substrate for microdomain physiology, it is not known if these channels regulate the spatiotemporal spread of signaling microdomains. Here, we employed a multiscale, morphologically realistic, conductance-based model of the hippocampal pyramidal neuron that accounted for experimental details of electrical and calcium-dependent biochemical signaling. We activated synaptic N-Methyl-d-Aspartate receptors through theta-burst stimulation (TBS) or pairing (TBP) and assessed microdomain propagation along a signaling pathway that included calmodulin, calcium/calmodulin-dependent protein kinase II (CaMKII) and protein phosphatase 1. We found that the spatiotemporal spread of the TBS-evoked microdomain in phosphorylated CaMKII (pCaMKII) was amplified in comparison to that of the corresponding calcium microdomain. Next, we assessed the role of two dendritically expressed inactivating channels, one restorative (*A*-type potassium) and another regenerative (*T*-type calcium), by systematically varying their conductances. Whereas *A*-type potassium channels suppressed the spread of pCaMKII microdomains by altering the voltage response to TBS, *T*-type calcium channels enhanced this spread by modulating TBS-induced calcium influx without changing the voltage. Finally, we explored cross-dependencies of these channels with other model components, and demonstrated the heavy mutual interdependence of several biophysical and biochemical properties in regulating microdomains and their spread. Our conclusions unveil a pivotal role for dendritic voltage-gated ion channels in actively amplifying or suppressing biochemical signals and their spatiotemporal spread, with critical implications for clustered synaptic plasticity, robust information transfer and efficient neural coding.

## Author summary

The spatiotemporal spread of biochemical signals in neurons and other cells regulate signaling specificity, tuning of signal propagation, along with specificity and clustering of adaptive plasticity. Theoretical and experimental studies have demonstrated a critical role for cellular morphology and the topology of signaling networks in regulating this spread. In this study, we add a significantly complex dimension to this narrative by demonstrating that voltage-gated ion channels on the plasma membrane could actively amplify or suppress the strength and spread of downstream signaling components. Given the expression of different ion channels with wide-ranging heterogeneity in gating kinetics, localization and density, our results point to an increase in complexity of and degeneracy in signaling spread, and unveil a powerful mechanism for regulating biochemical-signaling pathways across different cell types.

## Introduction

Microdomains that emerge from spatially constricted spread of biochemical signaling components play a central role in defining several neuronal computations, including compartmentalization of neuronal plasticity and localized targeting of membrane components [1–10]. Theoretical and experimental studies have demonstrated that the spread of these microdomains are regulated by several biophysical and biochemical parameters. These parameters include the concentrations, localization profiles, binding and diffusion constants of the signaling components that are part of the signaling network, the morphological structure of the compartment, network topologies and feedback motifs [1–5,8,11]. Most such studies have considered neuronal dendrites, which form the prominent structural substrate for microdomain spread and physiology, to be passive structures that lack active dendritic conductances. Neuronal electrical signaling and physiology, however, is defined by the presence and plasticity of voltage-gated ion channel conductances, some of which are present at higher densities in the dendrites than at the cell body [9,12–24]. Additionally, consequent to their ability to significantly alter calcium influx into neuronal compartments, these active dendritic conductances are well established as critical regulators of synaptic plasticity profiles [12–14,19,25–35]. Given such pivotal role of active dendrites in determining neuronal physiology and plasticity, the question on whether active dendritic conductances regulate the spatiotemporal spread of signaling microdomains is important and has not been addressed.

To address this question, we employed a multiscale, multicompartmental, morphologically realistic, conductance-based model that accounted for the biophysics of electrical signaling [16–18,36,37] and the biochemistry of calcium handling [25,26,38–40] and downstream enzymatic signaling in a hippocampal pyramidal neuron. We chose the calcium–calmodulin–calcium/calmodulin-dependent protein kinase II (CaMKII)–protein phosphatase 1 (PP1) signaling pathway owing to its critical importance to several forms of neuronal plasticity [41–52], and employed physiologically relevant theta-burst stimulation (TBS) or theta-burst pairing (TBP) protocol [12,20,53–58] to initiate a calcium microdomain through N-Methyl-d-Aspartate receptor (NMDAR) activation at a synapse. We studied the spatiotemporal spread of calcium and other downstream microdomains in a dendritic segment compartmentalized to 2000 compartments, each spanning ~97-nm of length.

Using this setup, we assessed the role of two dendritically expressed inactivating conductances [16,18]—one restorative (*A*-type K^+^) and another regenerative (*T*-type Ca^2+^)—and showed that they respectively suppress and enhance the spread of phosphorylated CaMKII through different mechanisms. We also assessed the cross-dependencies of these two channels with other model components, and demonstrated the heavy mutual interdependence of several biophysical and biochemical properties in regulating microdomains and their spread. Our results provide compelling evidence for a critical role of active dendrites in regulating the spatiotemporal spread of signaling microdomains. These conclusions call for a marked rethink of the complexities associated with subcellular signaling networks, with future experiments focusing on the role of voltage-gated ion channels in tuning location-dependent signaling specificity and spread, in regulating robust information transfer and efficient encoding of afferent inputs in signaling networks, in regulating clustered plasticity of spatially-adjacent synapses on dendritic branches, and in behavioral state- and activity-dependent changes in such signal propagation.

## Results

**Fig 1:**
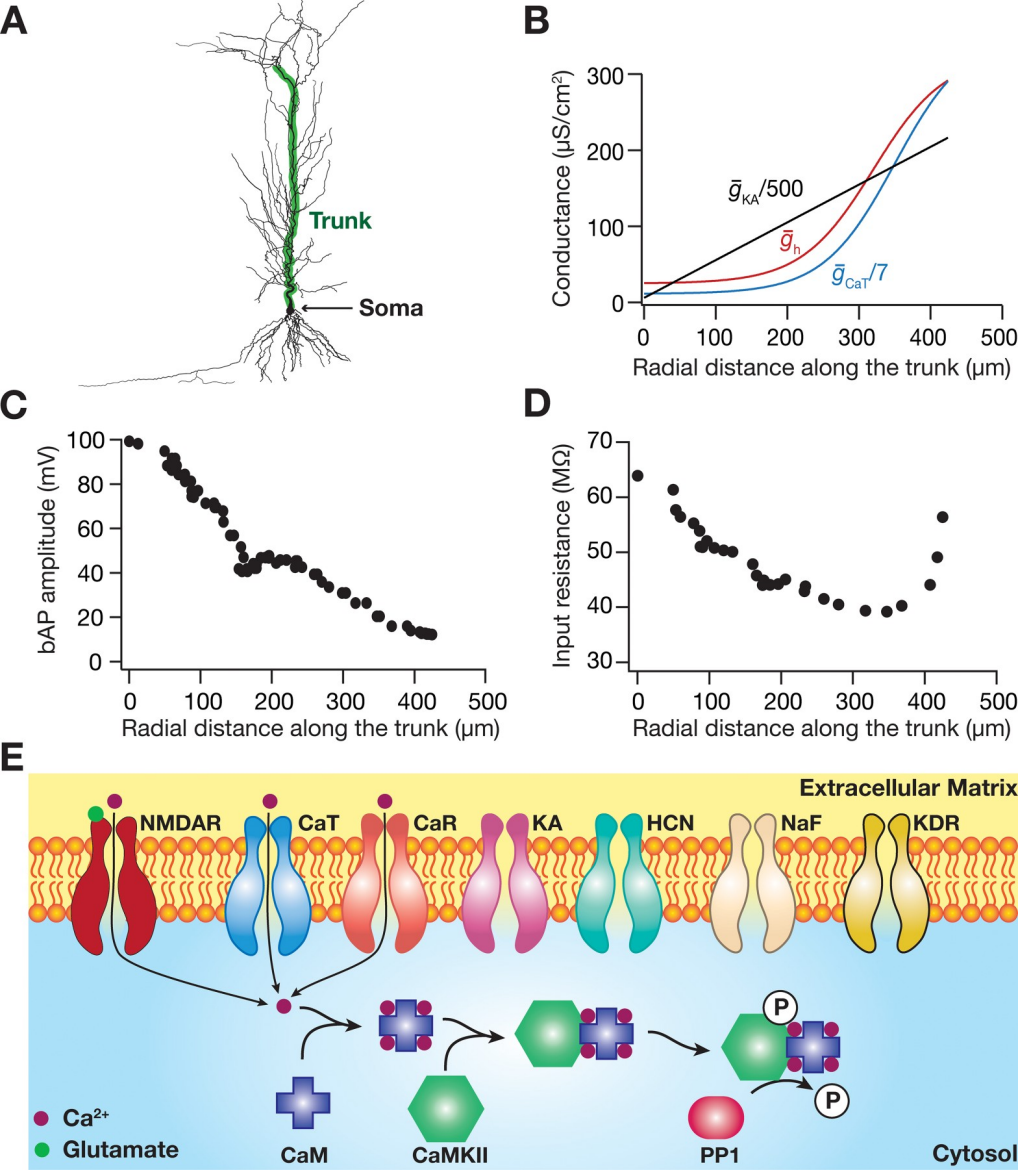
Functional maps in the morphologically realistic base model match with experimental observations. (*A*) The morphology of a CA1 pyramidal model neuron with the trunk (along which the measurements in *B–D* are plotted) highlighted. (*B*) Gradients in somatodendritic expression profile of three channels: *A*-type K^+^ (${\bar{g}}_{KA}$), *T*-type Ca^2+^ (${\bar{g}}_{CaT}$) and hyperpolarization-activated cyclic-nucleotide-gated nonspecific-cation, HCN (${\bar{g}}_{h}$) channels. (*C–D*) Backpropagating action potential (bAP) amplitude (*C*) and input resistance (*D*) plotted as function of distance from the soma along the somatoapical trunk. (*E*) Schematic representation of the signaling pathway that was assessed in the study, also depicting the various channels whose impacts on the spread of signaling microdomains were analyzed. NMDAR: *N*-Methyl d-Aspartate receptors; CaT: *T*-type calcium channel; CaR: *R*-type calcium channel; KA: *A*-type potassium channel; HCN: hyperpolarization-activated cyclic-nucleotide-gated nonspecific-cation channel; NaF: fast sodium channel; KDR: delayed rectifier potassium channel; CaM: calmodulin; CaMKII: calcium/calmodulin-dependent protein kinase II; PP1: protein phosphatase. The baseline active ion channel conductances with increasing radial distance along the trunk (highlighted) varied as: ${\bar{g}}_{NaF}$ = 16 mS/cm^2^ throughout, ${\bar{g}}_{KDR}$ = 10 mS/cm^2^ throughout, ${\bar{g}}_{KA}$ = 3.1–108.35 mS/cm^2^ (Eq 3), ${\bar{g}}_{h}$ = 25–291 μS/cm^2^ (Eq 4), ${\bar{g}}_{CaT}$ = 0.08–2.03 mS/cm^2^ (Eq 5), ${\bar{g}}_{CaR}$ = 0.

Neuronal excitability is critically regulated by morphological as well as intracellular channel localization profiles [13–15,19,59–61]. As arbitrary choices for morphological properties and channel parameters would preclude extrapolations of our results to physiology, as a first step, we employed a hippocampal pyramidal neuron reconstruction and systematically matched the electrical properties of this model neuron with their electrophysiological counterparts (Fig 1*A–*1*D*). This was especially important because a goal of the study was to assess the dependence of signaling spread on neuronal excitability, therefore necessitating the excitability of the model to match measurements from hippocampal neurons across the somatodendritic arbor. The specific signaling pathway that we chose to assess in this study is depicted in Fig 1*E*. The rationale behind the choice of this pathway was its critical importance to several forms of neuronal plasticity [41–52]. Additionally, from a physiological standpoint, the spread of the CaMKII microdomain in this signaling pathway directly translates to the spread of neuronal plasticity through phosphorylation of several substrates that include 2-amino-3-(5-methyl-3-oxo-1,2-oxazol-4-yl) propanoic acid (AMPA) receptors and several ion channels [4,42,43,51,52,56,62]. As the assessment of microdomains requires the analyses of the spatiotemporal spread of each signaling component across the neuronal structure [4,7,8], we employed partial differential equations to model the reaction kinetics coupled to the diffusion of the individual components in the signaling pathway.

### Theta burst stimulation to a synapse evoked localized microdomains of calcium and downstream signaling components

**Fig 2:**
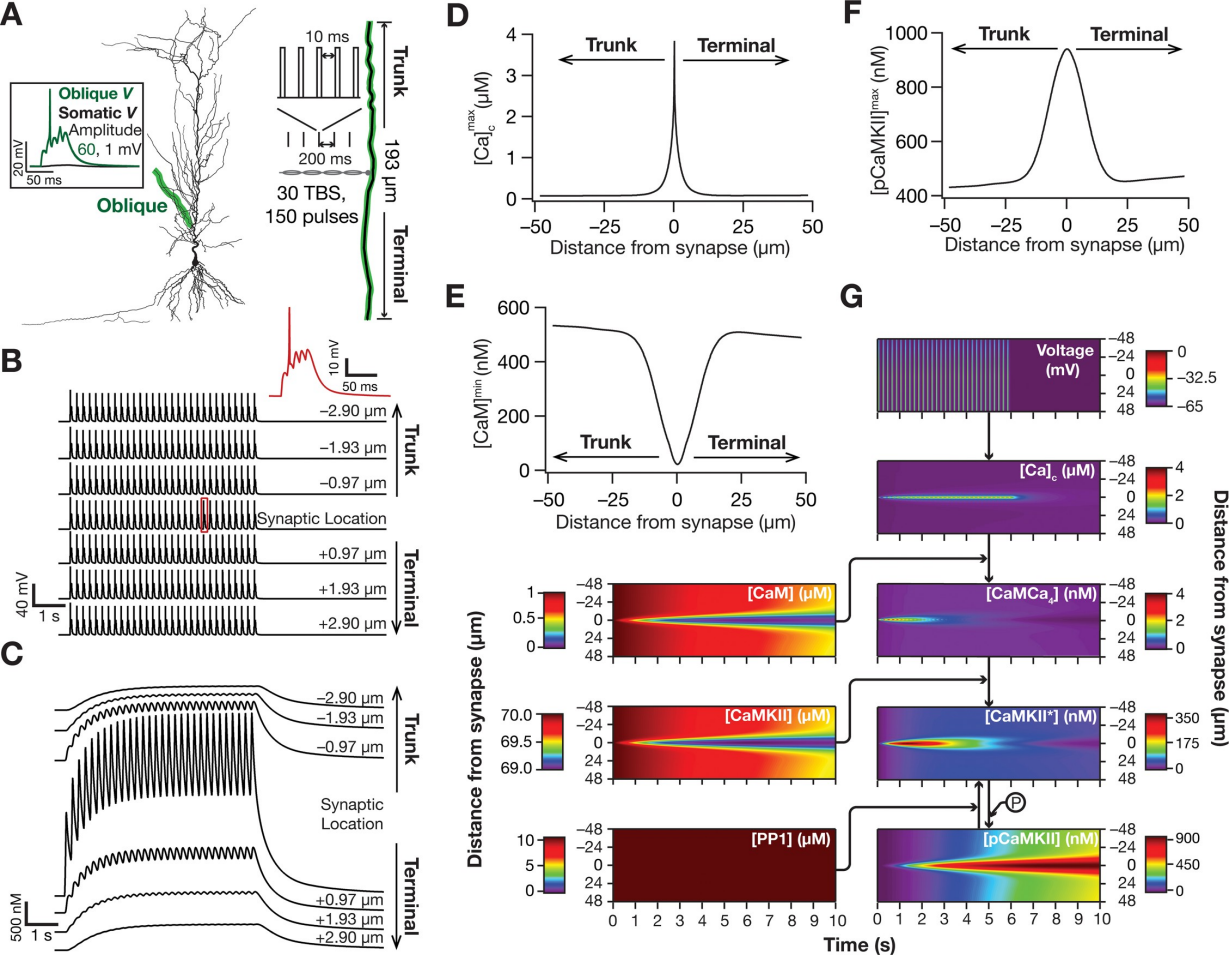
Theta burst stimulation to a synapse evoked localized microdomains of calcium and phosphorylated CaMKII. (*A*) *Left*, The morphology of the model neuron with the highlighted oblique where the synapse was located. All traces and analyses presented in *B–G* were from this oblique dendrite. *Right*, The position of the synapse within this oblique is represented in a zoomed version. Also shown is the theta burst stimulus (TBS) protocol where 30 bursts, each made of 5 synaptic stimulation pulses (intra-burst frequency: 100 Hz), were presented at 5 Hz. Across all figures depicting distances from the synapse, to avoid ambiguity, distances towards terminal and trunk are represented as positive and negative numbers, respectively. *Inset*, Voltage response at the center of the oblique (green) where the synapse is located and simultaneously recorded somatic (black) voltage response to a single burst of the TBS stimulus (100 Hz, five pulses). It may be noted that the local oblique response manifests a dendritic spike (amplitude of voltage response at oblique: 60 mV), which does not propagate to the cell body (amplitude of voltage response at soma: 1 mV) owing to attenuation during its somatodendritic propagation. (*B*) Voltage responses to TBS recorded at the synaptic location and at different distances, towards the trunk and the terminal, from this location. Trace in red: Voltage response to a single burst of synaptic stimulation, which is highlighted in red on the trace recorded at the synaptic location. (*C*) Cytosolic calcium [*Ca*]_c_ traces recorded in response to TBS, at the same locations as that of the voltage response in *B*. (*D–F*) Maximum value of cytosolic calcium concentration, ${\left[Ca\right]}_{c}^{max}$ (*D*), minimum value of unbound calmodulin concentration, [*CaM*]^min^ (*E*) and maximum value of phosphorylated CaMKII concentration, [*pCaMKII*]^max^ (*F*) plotted as functions of distance from the synapse. A double exponential fit (of the form *A* + *B* exp(−*x*/*λ*_1_) + *C* exp(−*x*/*λ*_2_)) to the distance-dependent calcium decay in panel (*D*) resulted in values for calcium space constants of *λ*_1_ = 0.1 μm and *λ*_1_ = 2 μm. (*G*) Kymograph flowchart of the signaling molecules showing the initiation (the calcium trace) and spatiotemporal propagation of microdomains along the signaling cascade. All parameters and channel conductances were assigned to their default values (Fig 1; Tables 1 and 2). Details of parametric values (of active and passive properties in the oblique dendrite shown in Fig 2*A*) employed for simulations presented in this figure are provided in S1 Table.

To accommodate the steep spatial decay of calcium, and the consequent requirement for finer spatial discretization when compared to discretization required for electrical simulations [63], we followed the established approach of employing a single oblique dendrite to assess signaling microdomains [8]. Although the analyses associated with signaling microdomains (Fig 2*A*) was performed in this oblique dendrite, its presence as part of the morphologically realistic electrical model ensured that the branching profiles and channel conductances required for maintaining excitability properties match their experimental counterparts. To accommodate fine spatial discretization, the oblique (of total length 193 μm) highlighted in Fig 2*A* was compartmentalized to 2000 compartments (making each compartment size to be around 97 nm). With the rest of the somatodendritic arbor discretized to accommodate electrical length constants, this spatial discretization procedure resulted in a total number of 2864 compartments in the entire neuronal structure.

We placed a single synapse containing both AMPA and NMDA receptors, whose kinetics and voltage-dependence properties (of NMDA receptors) were derived from electrophysiological measurements, at the center of this 100-μm region on the oblique. The AMPAR density was set such that the somatic unitary EPSP amplitude was ~0.2 mV, to match with experimental observations [64]. Consistent with our motivations of understanding signaling microdomains that are relevant to plasticity induction in the hippocampus, we stimulated this synapse with the well-established theta burst stimulation (TBS; Fig 2*A*) protocol that induces synaptic plasticity in hippocampal neurons [53]. As expected, the voltage traces obtained with this stimulation resulted in temporally summating excitatory postsynaptic potentials (EPSPs) that resulted in local dendritic, but not axosomatic spikes (Fig 2*A*). Although the recorded voltage traces were not very different across a span of 3 μm on either side of the synapse (Fig 2*B*), the calcium concentration (consequent to influx through NMDARs at the synapse) displayed sharp attenuation with distance, thereby establishing the calcium microdomain induced by TBS (Fig 2*C and* 2*D*).

Given the reaction-diffusion framework employed here (Fig 1*E*), this calcium microdomain propagated along the signaling network, manifesting as localized increases in the concentration of calcium-bound calmodulin, activated and phosphorylated CaMKII (pCaMKII) and as localized reductions in the concentrations of unbound calmodulin and non-activated CaMKII (Fig 2*E*–2*G*). As would be expected from the binding kinetics of the reactions, and especially by the autophosphorylation of CaMKII, there was an increase in the spatiotemporal spread of the microdomain associated with pCaMKII compared to that of calcium (compare Fig 2*D* with Fig 2*F*; see Fig 2*G*). These results quantitatively demonstrate that TBS induces localized calcium influx, which, through propagation along an established signaling pathway, results in a microdomain of pCaMKII with a spread larger than that of the calcium microdomain [41,43,47].

**Fig 3:**
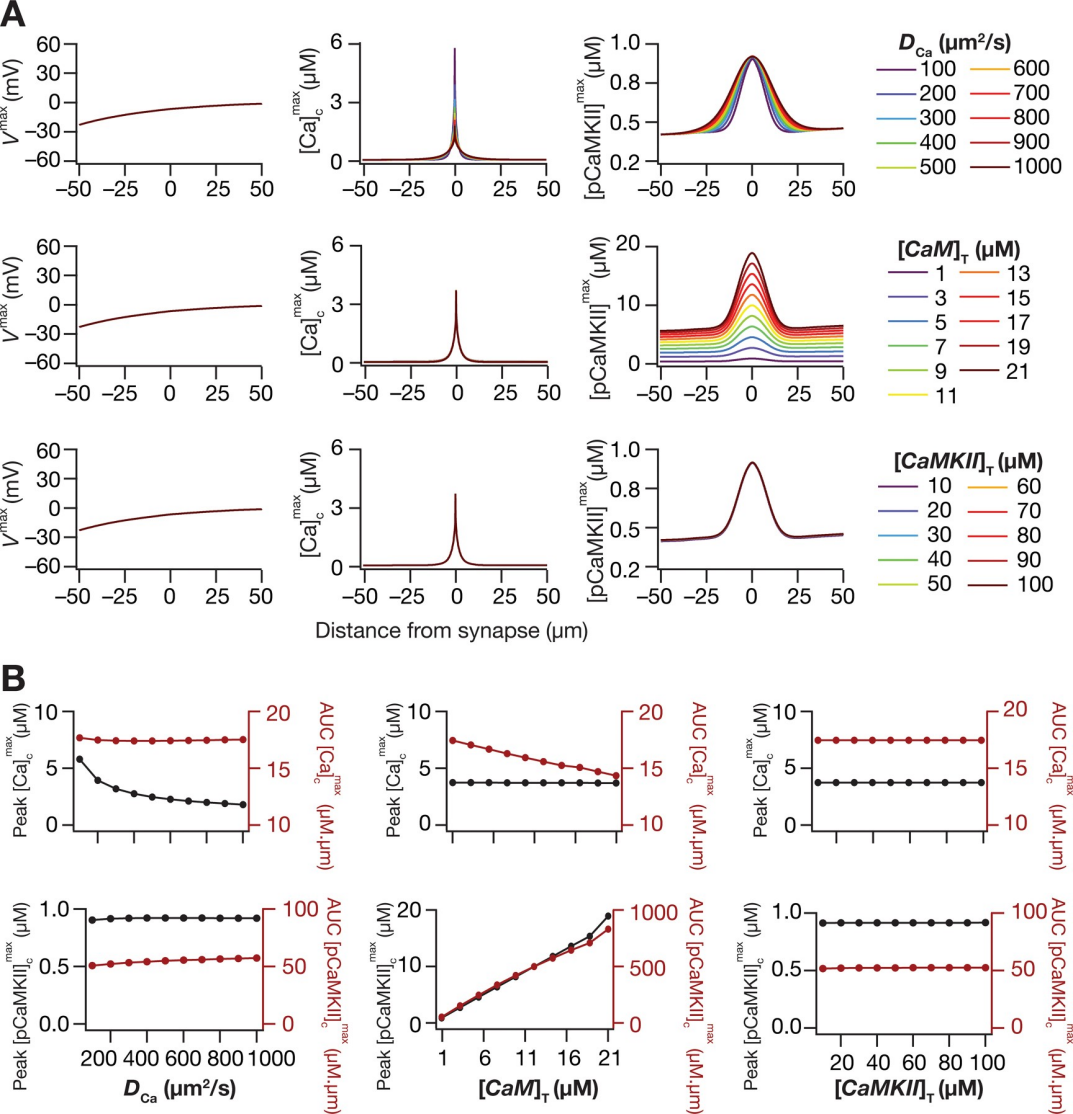
Sensitivity analyses on the impact of calcium diffusion constant and total concentrations of calmodulin and CaMKII concentrations on microdomains. (*A*) Sensitivity analyses depicting the effect of calcium diffusion constant, *D*_Ca_ (top), total CaM concentration, [*CaM*]_T_ (middle) and total CaMKII concentration, [*CaMKII*]_T_ (bottom) levels on peak voltage, *V*^max^ (left), maximum value of cytosolic calcium concentration, ${\left[Ca\right]}_{c}^{max}$ (middle) and maximum value of phosphorylated CaMKII concentration, [*pCaMKII*]^max^ (right) as recorded in response to TBS. (*B*) Peak value (black) and area under the curve, AUC (red) of ${\left[Ca\right]}_{c}^{max}$ (top panels) and [*pCaMKII*]^max^ (bottom panels) plotted against increasing *D*_Ca_ (left), [*CaM*]_T_ (middle) and [*CaMKII*]_T_ (right). Except for parameters that were altered, all other parameters and channel conductances were assigned to their default values (Fig 1; Tables 1 and 2). Details of parametric values (of active and passive properties in the oblique dendrite shown in Fig 2*A*) employed for simulations presented in this figure are provided in S1 Table.

In order to look at how several key parameters of the model affect the micorodomain spatiotemporal kinetics, we performed sensitivity analyses involving different values of calcium diffusion constant (*D*_Ca_), total concentrations of calmodulin ([*CaM*]_T_) and CaMKII ([*CaMKII*]_T_). We observed no significant change in the electrical response to TBS for different values assigned to each of these three parameters (Fig 3*A*), which was expected because all these parameters are involved in the signaling pathway downstream of electrical responses. However, the spatiotemporal dynamics of downstream signaling microdomains were sensitive to these parametric values (Fig 3*A*). Specifically, increase in *D*_Ca_ expectedly enhanced the spatial spread, also resulting in a reduction in peak calcium concentration (Fig 3*A and* 3*B*). Consequent to the enhanced spatial spread of calcium, the pCaMKII microdomains showed an enhanced spread, although there was little increase in peak [*pCaMKII*]^max^ (Fig 3*A* and 3*B*). Upon increase in [*CaM*]_T_, as more free calcium was now bound to CaM, there was a small reduction in the peak values of ${\left[Ca\right]}_{c}^{max}$ (Fig 3*A and* 3*B*). As a direct consequence of the larger availability of calcium-bound calmodulin, there was a significant increase in the peak values of [*pCaMKII*] and in the spread of pCaMKII (Fig 3*A and* 3*B*). Varying the [*CaMKII*]_T_, however, did not alter the calcium or pCaMKII dynamics significantly across the tested range (Fig 3*A* and 3*B*). These results demonstrated that within the specific parametric configurations, the spatiotemporal evolution of pCaMKII was more sensitive to the concentration of calmodulin than on calcium diffusion or on total CaMKII concentration.

### The presence of *A*-type potassium channels suppressed the spread of microdomains across the signaling pathway

**Fig 4:**
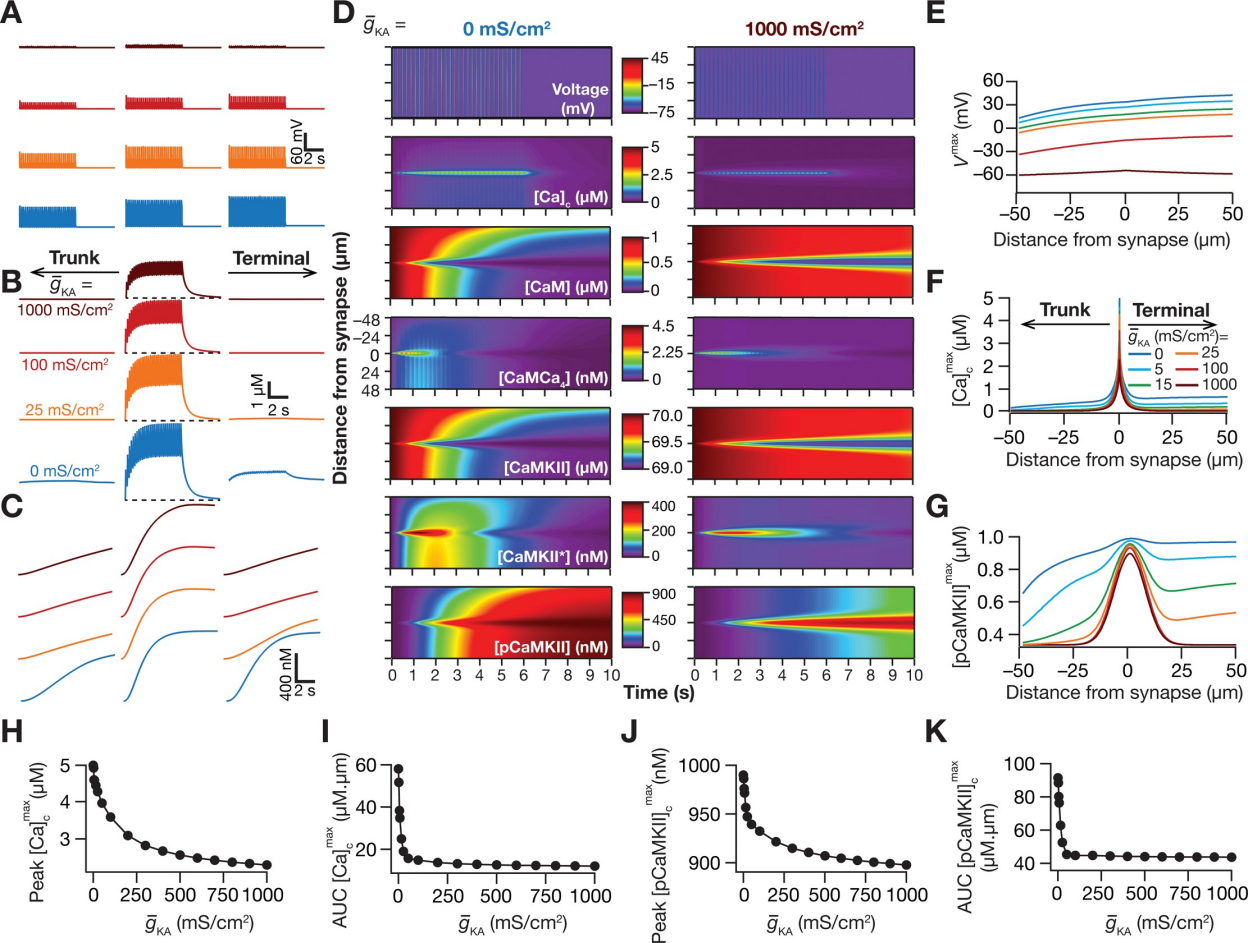
*A-*type potassium channels suppressed the spread of signaling microdomains in all species of the signaling network. (*A–C*) Voltage (*A*), cytosolic calcium (*B*) and phosphorylated CaMKII (*C*) traces recorded, at different locations along the oblique for different values of *A*-type K^+^ channel conductance ${\bar{g}}_{KA}$, in response to theta-burst stimulation. The center traces were recorded at the synaptic location, whereas the plots on the left and right were from compartments that were 50 μm from the synaptic location, toward the trunk and terminal, respectively. The dashed black lines in panel *B* represent the initial resting calcium concentration (50 nM). (*D*) Kymographs of the signaling molecules showing the initiation (the calcium trace) and spatiotemporal propagation of microdomains along the signaling cascade, in response to identical TBS with two different representative values of the *A*-type potassium channel conductance: ${\bar{g}}_{KA}$ = 0 mS/cm^2^ (left) and 1000 mS/cm^2^ (right). (*E*–*G*) Peak voltage, *V*^max^ (*E*), maximum value of cytosolic calcium concentration, ${\left[Ca\right]}_{c}^{max}$ (*F*) and maximum value of phosphorylated CaMKII concentration, [*pCaMKII*]^max^ (*G*) plotted as functions of distance from the synapse. (*H*–*I*) Peak value (*H*) and area under the curve, AUC (*I*) of ${\left[Ca\right]}_{c}^{max}$ plotted against ${\bar{g}}_{KA}$. *J–K*, Peak value (*J*) and AUC (*K*) of [*pCaMKII*]^max^ plotted against ${\bar{g}}_{KA}$. Note that the AUC values presented in *I* and *K* correspond to representative traces shown in *F* and *G*. For simulations in this figure, the conductance values for the oblique represented in Fig 2*A* were: ${\bar{g}}_{CaR}$ = 100 mS/cm^2^ and ${\bar{g}}_{h}={\bar{g}}_{CaT}$ = 0 mS/cm^2^. All other compartments were assigned to default values of conductances (Fig 1). Details of parametric values (of active and passive properties in the oblique dendrite shown in Fig 2*A*) employed for simulations presented in this figure are provided in S1 Table.

The presence and plasticity of *A*-type potassium (KA) channels in hippocampal neuronal dendrites and their roles in regulating dendritic excitability and synaptic plasticity profiles are well established [16,20–22,25–27,29,30,65–68]. Does the presence of or plasticity in KA channels in hippocampal dendrites alter the spread of microdomains in plasticity-inducing enzymes? To address this question, we performed the simulations described in Fig 2 with different densities of KA channels in the oblique specified in Fig 2*A* (see S1 Table for a figure-wise catalog of oblique parameters). As would be expected from the ability of KA channels to regulate synaptic and action potential amplitudes [16], we found that the voltage response to TBS was lower when the density of KA channels was increased (Fig 4*A*, 4*D and* 4*E*). This difference in voltage response directly translated to changes in the calcium influx through NMDARs (Fig 4*B*, 4*D*, 4*F*, 4*H and* 4*I*), and introduced small changes in the spread of the calcium microdomain, especially towards the terminal (Fig 4*D* and 4*F*).

Strikingly, this small increase in the spread of calcium microdomain was significantly amplified with propagation along the signaling pathway (Fig 4*D*, 4*F* and 4*G*). Specifically, although the peak pCAMKII response was not very different across different densities of KA channels (Fig 4*G* and 4*J*), the spread of the pCaMKII microdomain showed tremendous enhancement with reduction in the density of KA channels (Fig 4*G* and 4*K*).

**Fig 5:**
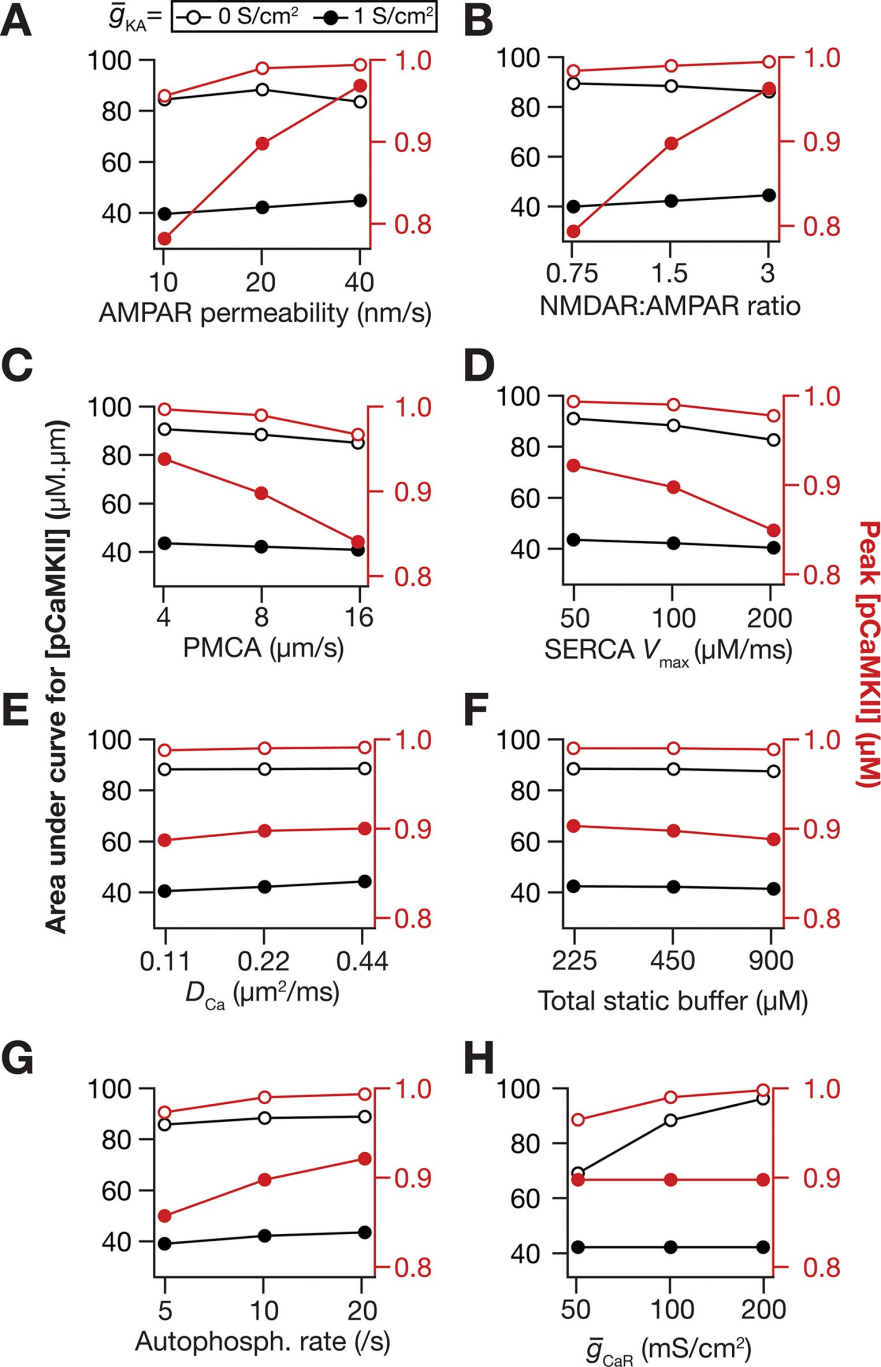
Sensitivity analyses spanning several model parameters confirmed a role for *A*-type potassium channels in suppressing microdomain spread. The impact of varying critical model parameter values on AUC (black) and peak values (red) of [*pCaMKII*]^max^ at ${\bar{g}}_{KA}$ = 0 S/cm^2^ (open circles) and ${\bar{g}}_{KA}$ = 1 S/cm^2^ (closed circles). (*A–H*) The parameters varied were AMPAR permeability (*A*), NMDAR:AMPAR ratio (*B*), plasma membrane calcium pump (PMCA) density (*C*), *V*_max_ of the SERCA pump (*D*), calcium diffusion constant, *D*_Ca_ (*E*), total static buffer concentration (*F*), CaMKII autophosphorylation rate (*G*), and *R*-type calcium channel conductance, ${\bar{g}}_{CaR}$ (*H*). For simulations in this figure, ${\bar{g}}_{CaT}={\bar{g}}_{h}$ = 0 mS/cm^2^ for the oblique represented in Fig 2*A*. Except for panel *H* where the sensitivity to ${\bar{g}}_{CaR}$ was assessed, the value of ${\bar{g}}_{CaR}$ used in these simulations was 100 mS/cm^2^. All other compartments were assigned to default values of conductances (Fig 1). Details of parametric values (of active and passive properties in the oblique dendrite shown in Fig 2*A*) employed for simulations presented in this figure are provided in S1 Table.

How sensitive are our conclusions to changes in key model parameters? To answer this question, we performed sensitivity analyses to assess the impact of KA channels on pCaMKII microdomain spread with two-fold increase or decrease in several key parameters (Fig 5). Across different parametric combinations, the peak pCaMKII and its spread were consistently larger with lower values of KA channel density, thereby confirming that our conclusions were not restricted by the choice of default parametric values. The sensitivity analyses also demonstrated that the spread of microdomains was critically reliant on several parameters [8], pointing towards robustness of microdomain spread through degeneracy involving several biochemical and biophysical components [8,69]. Specifically, an increase in synaptic AMPAR (Fig 5*A*) or NMDAR (Fig 5*B*) densities, or the calcium diffusion constant (Fig 5*E*), or the autophosphorylation rate of CaMKII (Fig 5*G*) or the density of *R*-type calcium (CaR) channels (Fig 5*H*) enhanced the spread of pCaMKII microdomain. In contrast, an increase in the rate associated with the plasma membrane calcium pump (Fig 5*C*) or the *V*_max_ of the SERCA pump (Fig 5*D*) or the total capacity of the calcium buffer (Fig 5*F*) suppressed the pCaMKII microdomain spread.

Together, these results unveil a pivotal role for dendritic *A*-type potassium channels in suppressing the spatiotemporal spread of microdomains in plasticity-inducing enzymes.

### The presence of *T*-type calcium channels enhanced the spread of microdomains across the signaling pathway

**Fig 6:**
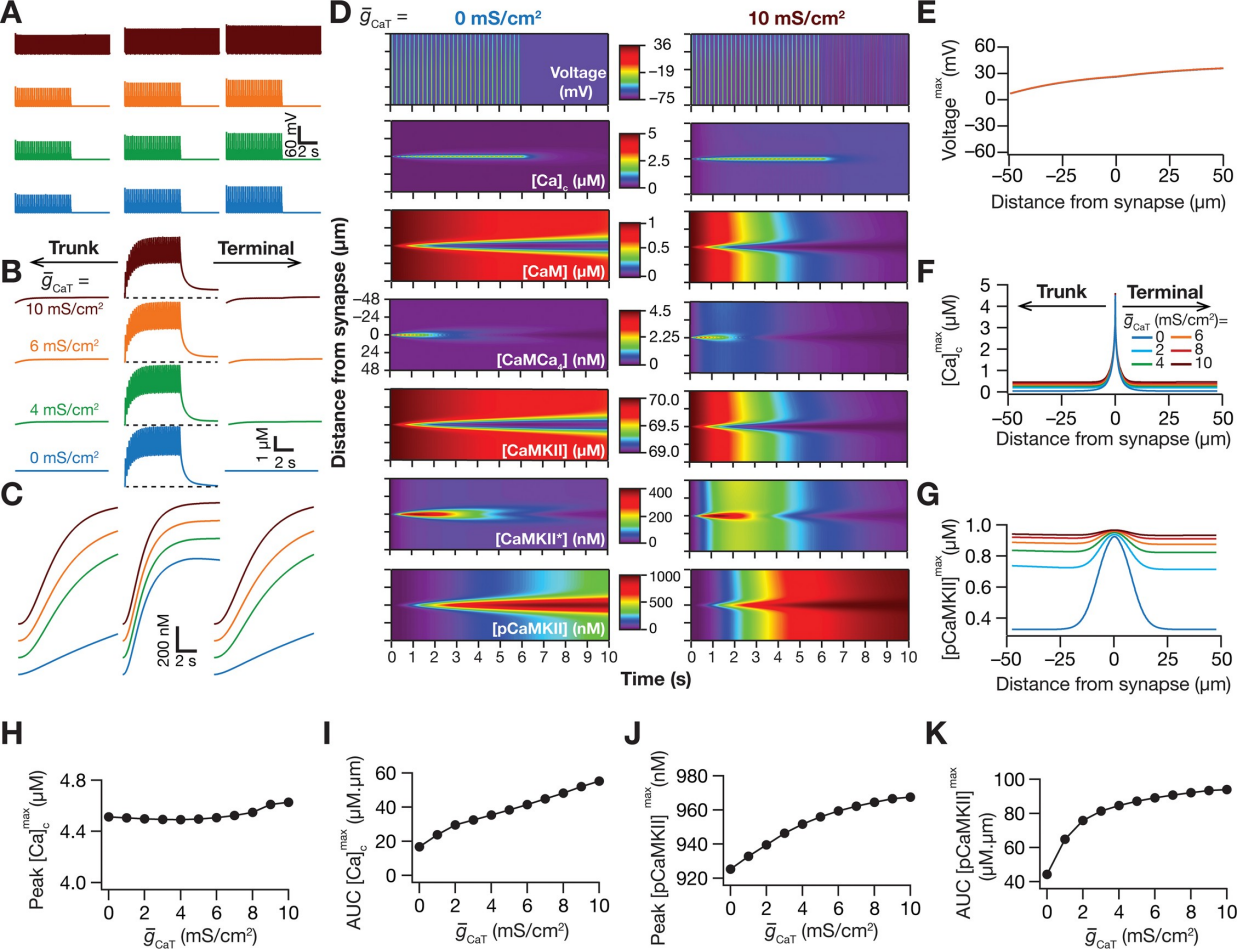
*T-*type calcium channels enhanced the spread of signaling microdomains in all species of the signaling network. (*A–C*) Voltage (*A*), cytosolic calcium (*B*) and phosphorylated CaMKII (*C*) traces recorded, at different locations along the oblique for different values of *T-*type calcium channel conductance (${\bar{g}}_{CaT}$), in response to theta-burst stimulation. The center traces were recorded at the synaptic location, whereas the plots on the left and right were from compartments that were 50 μm from the synaptic location, toward the trunk and terminal, respectively. The dashed black lines in panel *B* represent the initial resting calcium concentration (50 nM), and it may be noted that at higher concentrations of CaT channels, the calcium concentration was higher than the resting concentration after the end of the TBS protocol. (*D*) Kymographs of the signaling molecules showing the initiation (the calcium trace) and spatiotemporal propagation of microdomains along the signaling cascade, in response to identical TBS with two different representative values of the *T*-type calcium channel conductance: ${\bar{g}}_{CaT}$ = 0 mS/cm^2^ (left) and 10 mS/cm^2^ (right). (*E*–*G*) Peak voltage, *V*^max^ (*E*), maximum value of cytosolic calcium concentration, ${\left[Ca\right]}_{c}^{max}$ (*F*) and maximum value of phosphorylated CaMKII concentration, [*pCaMKII*]^max^ (*G*) plotted as functions of distance from the synapse. (*H*–*I*) Peak value (*H*) and area under the curve, AUC (*I*) of ${\left[Ca\right]}_{c}^{max}$ plotted against ${\bar{g}}_{CaT}$. *J–K*, Peak value (*J*) and AUC (*K*) of [*pCaMKII*]^max^ plotted against ${\bar{g}}_{CaT}$. Note that the AUC values presented in *I* and *K* correspond to representative traces shown in *F* and *G*. For simulations in this figure, the conductance values for the oblique represented in Fig 2*A* were: ${\bar{g}}_{CaR}={\bar{g}}_{h}={\bar{g}}_{KA}$ = 0 mS/cm^2^. All other compartments were assigned to default values of conductances (Fig 1). Details of parametric values (of active and passive properties in the oblique dendrite shown in Fig 2*A*) employed for simulations presented in this figure are provided in S1 Table.

Low voltage-activated transient *T*-type calcium (CaT) channels, with their predominant dendritic presence, significantly alter synaptic integration, calcium influx and dendritic spike initiation in hippocampal pyramidal neurons [18,70,71]. Although the diverse roles of CaT channels in regulating neuronal physiology and plasticity have been explored [72–76], it is not known if these channels contribute to the spread of signaling microdomains of plasticity-inducing enzymes. Therefore, as a next step, we repeated the simulations described in Fig 2 with different densities of CaT channels in the oblique specified in Fig 2*A*. We found that the peak local voltage response to TBS was slightly higher at terminal end. Additionally, the spatiotemporal voltage response profile was not significantly different with various densities of CaT channels (Fig 6*A* and 6*E*), although the cell entered spontaneous spiking with very high density of these channels (*e*.*g*., Fig 6*A*; top panel). Although the peak calcium response was not very different with different densities of CaT channels (Fig 6*B*, 6*F* and 6*H*), there was a small increase in the spread of calcium microdomain with increase in CaT-channel density (Fig 6*B*, 6*F* and 6*I*).

Despite the absence of large changes in peak voltage and calcium and pCaMKII (Fig 6*C*, 6*G* and 6*J*) responses at the location of the synapse, we found a significantly large increase in the spread of the pCaMKII microdomain with increase in CaT-channel density (Fig 6*C*, 6*G* and 6*K*). Additionally, and in contrast to the case with KA channels (Fig 4*G*), this increase in spread was symmetric about the synaptic location, which was a consequence of the manner in which these channels altered the microdomain spread. Specifically, whereas KA channels altered the spread of microdomains by asymmetrically modulating the voltage and calcium responses (Fig 4*E and* 4*F*), CaT channels modulated the spread through symmetric changes in calcium spread (Fig 6*F*) without significant changes in voltage response (Fig 6*E*).

**Fig 7:**
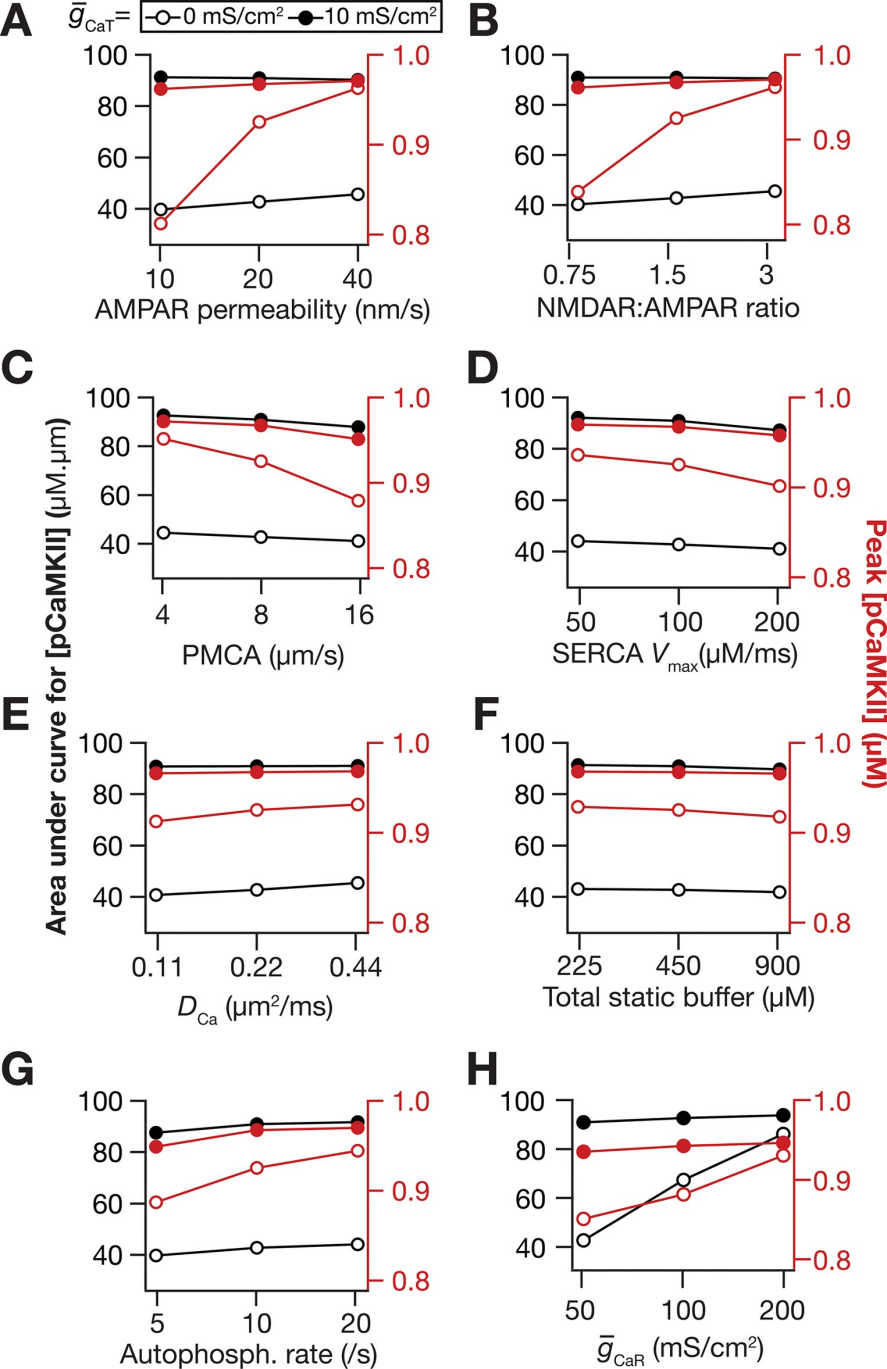
Sensitivity analyses spanning several model parameters confirmed a role for *T*-type calcium channels in enhancing microdomain spread. The impact of varying critical model parameter values on AUC (black) and peak values (red) of [*pCaMKII*] at ${\bar{g}}_{CaT}$ = 0 mS/cm^2^ (open circles) and ${\bar{g}}_{CaT}$ = 10 mS/cm^2^ (closed circles). (*A–H*) The parameters varied were AMPAR permeability (*A*), NMDAR:AMPAR ratio (*B*), plasma membrane calcium pump (PMCA) density (*C*), *V*_max_ of the SERCA pump (*D*), calcium diffusion constant, *D*_Ca_ (*E*), total static buffer concentration (*F*), CaMKII autophosphorylation rate (*G*), and *R*-type calcium channel conductance, ${\bar{g}}_{CaR}$ (*H*). For simulations in this figure, the conductance values for the oblique represented in Fig 2*A* were: ${\bar{g}}_{h}={\bar{g}}_{KA}$ = 0 mS/cm^2^. Except for panel *H* where the sensitivity to ${\bar{g}}_{CaR}$ was assessed, the value of ${\bar{g}}_{CaR}$ used in these simulations was 0 mS/cm^2^. All other compartments were assigned to default values of conductances (Fig 1). Details of parametric values (of active and passive properties in the oblique dendrite shown in Fig 2*A*) employed for simulations presented in this figure are provided in S1 Table.

Next, we performed sensitivity analyses on our model to assess the impact of CaT channels on pCaMKII microdomain spread with two-fold increase or decrease in several key parameters (Fig 7). We found, across different parametric combinations, that the spread of pCaMKII was consistently larger with higher values of CaT channel density, thereby confirming that our conclusions were not restricted by the choice of default parametric values. Results from these sensitivity analyses with reference to individual parameters (Fig 7) corroborated our earlier conclusions (Fig 5) on their specific roles in altering pCaMKII microdomains, also providing further evidence for degeneracy in the spatiotemporal spread of signaling microdomains.

Together, our results provide compelling evidence for a critical role for dendritic *T*-type calcium channels in enhancing the spatiotemporal spread of microdomains in plasticity-inducing enzymes, brought about by increases in calcium influx through these channels.

### Joint regulation of signaling microdomains by *A*-type potassium and *T*-type calcium channels

**Fig 8:**
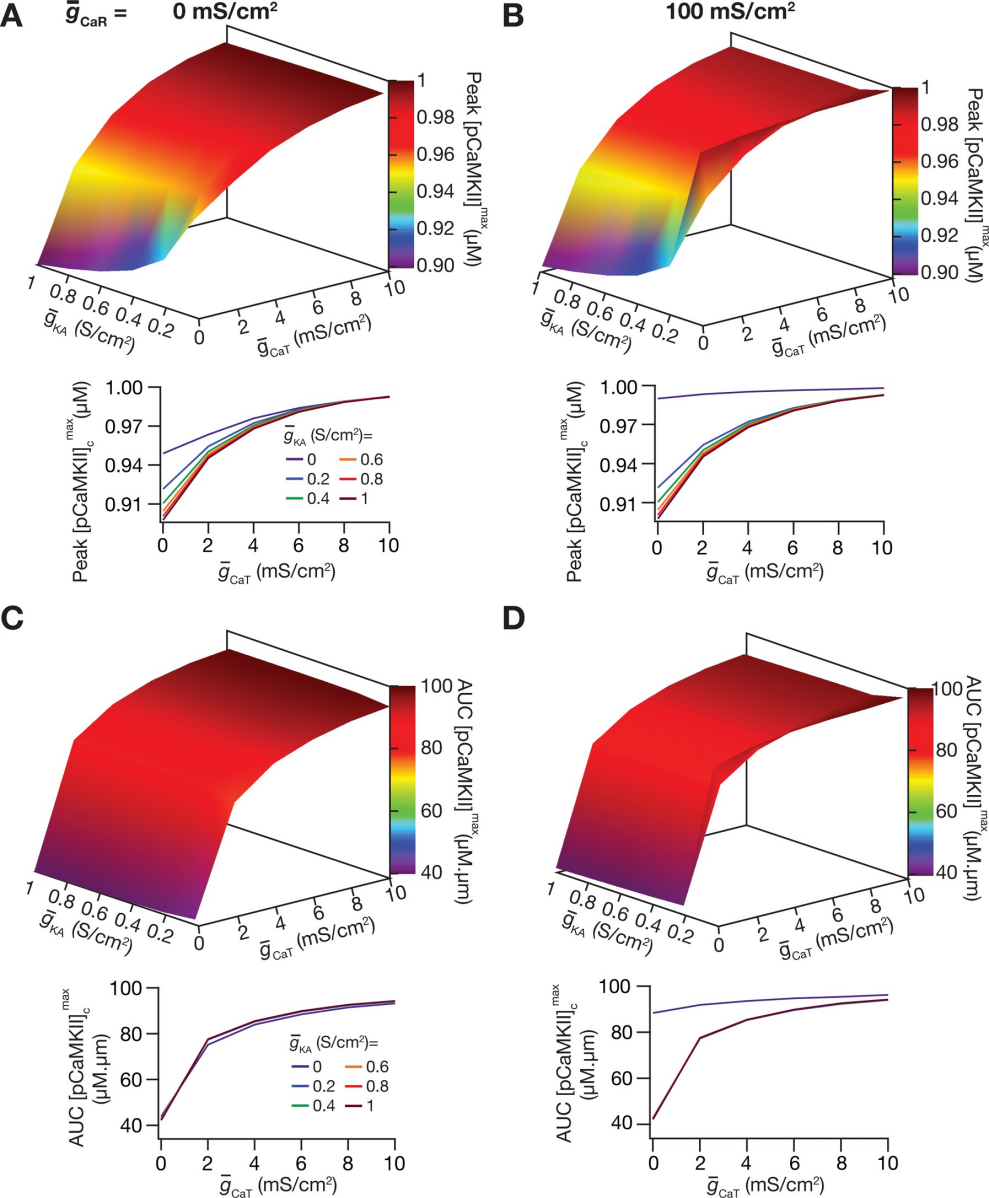
Analyses performed in the presence of both *T*-type calcium and *A*-type potassium channels confirmed the contrasting roles of these channels in regulating the spatiotemporal spread of signaling microdomains. (*A*–*B*) Plots depicting the peak values of [*pCaMKII*]^max^ with different ${\bar{g}}_{KA}$ and ${\bar{g}}_{CaT}$ values at ${\bar{g}}_{CaR}$ = 0 mS/cm^2^ (*A*) and ${\bar{g}}_{CaR}$ = 100 mS/cm^2^. (*B*). The graphs in the bottom panels represent the data presented in the top panel, but plotted differently. (*C*–*D*) Same as *A–B*, but for AUC of [*pCaMKII*]^max^. For simulations in this figure, all conductances (other than the ones specified for the oblique under consideration) across compartments were let to assume their respective default values. The oblique ${\bar{g}}_{h}$ was set to 0 for this set of simulations. Details of parametric values (of active and passive properties in the oblique dendrite shown in Fig 2*A*) employed for simulations presented in this figure are provided in S1 Table.

As a next step in our analyses, instead of individually varying either KA (Figs 4 and 5) or CaT (Figs 6 and 7) channel densities, we varied both channels together in the oblique to different densities and repeated our simulations to assess the spread of pCaMKII microdomains (Fig 8). We performed these simulations at two different densities of CaR channels to assess the interactions between KA, CaT and CaR channels in regulating microdomains. Although results from this set of simulations were consistent with our overall conclusions that KA and CaT channels respectively suppress and enhance the pCaMKII microdomain spread, the quantitative changes observed were dependent on the other channels present in the dendritic branch. For instance, when CaR channels were absent, the impact of KA channels on pCaMKII and its spread (Fig 8*A* and 8*C*) was minimal compared to that when CaR channels were present (Fig 8*B* and 8*D*). In contrast, from the same set of figures, it may be noted that the impact of CaT channels on pCaMKII and its spread was minimal when CaR channels were absent than when they were present (especially when KA channels were absent). Together these results unveil a crucial role for active dendritic conductances in regulating the spatiotemporal spread of signaling microdomains. Additionally, in conjunction with the sensitivity analyses presented earlier (Fig 3, Fig 5, Fig 7), our conclusions underscore the heavy mutual interdependence of several biophysical and biochemical properties (that account for synaptic, intrinsic and kinetic parameters of several membrane and cytosolic signaling components) in regulating microdomains and their spatiotemporal spread along a signaling pathway.

### Spatiotemporal spread of signaling microdomains when synapse was localized on a spine and in the presence of background synaptic activity

**Fig 9:**
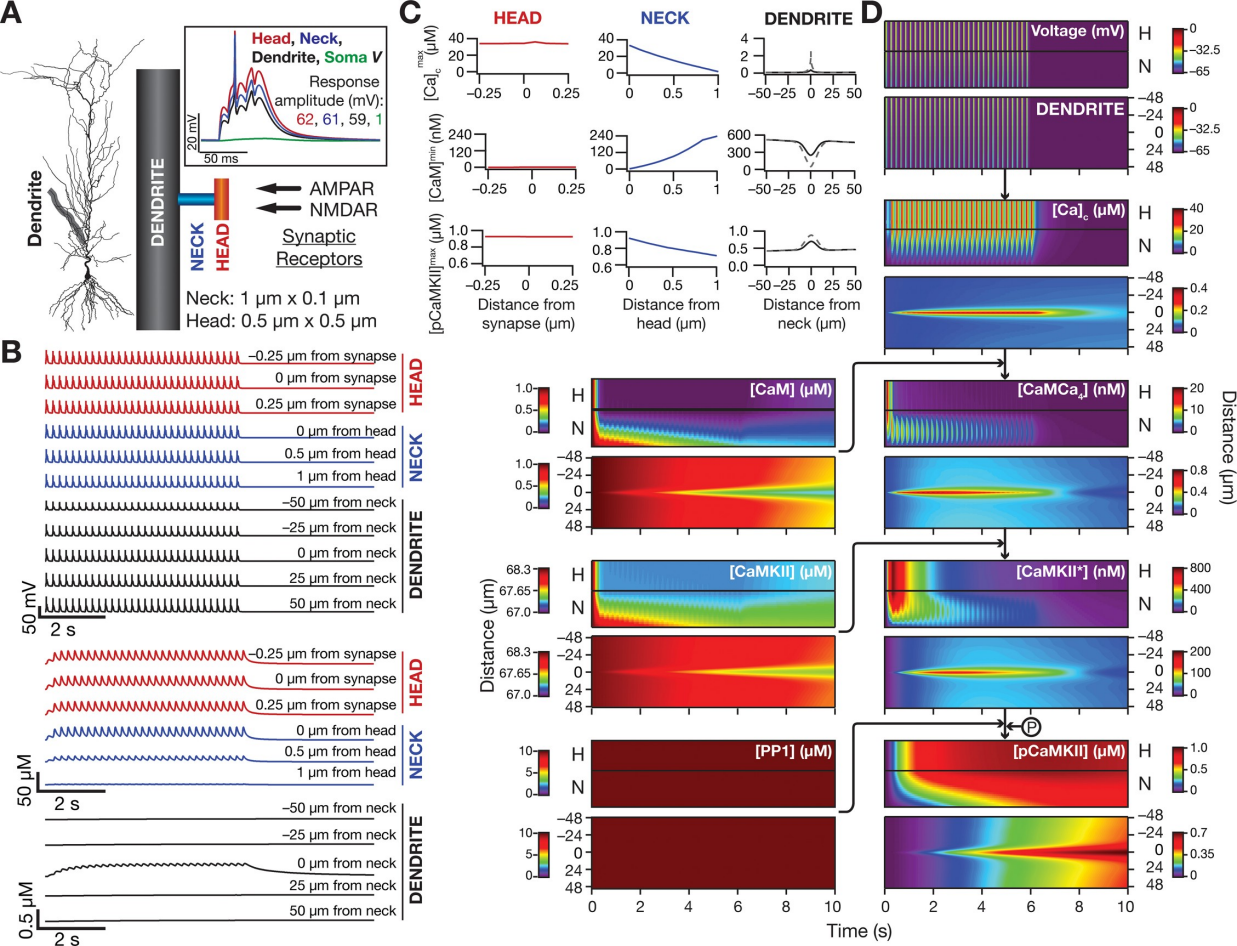
Impact of spine localization of the synapse on dendritic microdomain spread. (*A*) Schematic showing a spine, made of a spine head compartment and a spine neck compartment, placed at the center of the chosen dendritic oblique (depicted in Fig 2*A*). A glutamatergic synapse with colocalized NMDAR and AMPAR was placed at the center of the spine-head, with the AMPAR density adjusted such that the unitary somatic response to the synaptic activation was ~0.2 mV. *Inset*, Simultaneously recorded voltage responses to a single burst of the TBS stimulus (100 Hz, five pulses) at the spine head (red), spike neck (blue), center of the oblique (black) where the spine is connected and at the soma (cyan). It may be noted that the local oblique response manifests a dendritic spike (amplitude of voltage response at oblique: 59 mV), which does not propagate to the cell body (amplitude of voltage response at soma: 1 mV) owing to attenuation during its somatodendritic propagation. (*B*) Example voltage (top 11) and calcium (bottom 11) traces from different locations of the spine-head (red), spine-neck (blue) and dendritic shaft (black) in response to theta burst stimulus, TBS. Note that different scale bars were employed to represent calcium responses at the head/neck and the shaft. (*C*) Maximum values of cytosolic calcium concentration, ${\left[Ca\right]}_{c}^{max}$ (top), minimum value of unbound calmodulin [*CaM*]^min^ (middle) and phosphorylated CaMKII concentration, [*pCaMKII*]^max^ (bottom) obtained in response to TBS, recorded simultaneously at various locations of the spine head (red), spine neck (blue) and dendritic shaft (black). Grey dashed lines in the dendritic graphs represent the maximal level of the same molecular species measured at the dendritic shaft in the absence of spine (from Fig 2). (*D*) Kymographs depicting the spatiotemporal spread of microdomains along the biochemical signaling cascade in response to TBS. The top panels depict the voltage/molecular concentrations at the 10 spine head compartments; the middle panels depict the same at the 20 spine neck compartments; the bottom panels depict kymographs for 200 dendritic compartments, plotted as spread from the location of the spine (50 μm on either side of the spine) on the dendrite. H: Spine head, N: Spine neck. All channel conductances were assigned to their baseline values as mentioned in Fig 1. Details of parametric values (of active and passive properties in the oblique dendrite shown in Fig 2*A*) employed for simulations presented in this figure are provided in S1 Table.

Excitatory synapses typically impinge on a dendritic spine, which has been postulated to form a biochemical compartment for calcium and downstream signaling molecules [4,51,77–87]. In order to study the effect of the presence of spine on the signaling microdomains, we incorporated a spine consisting of a head and a neck on the dendritic shaft (Fig 9*A*) and recorded the spread of voltage, calcium and the downstream signaling molecules in all the three sections. The AMPAR density in the spine was set such that the somatic unitary EPSP amplitude was ~0.2 mV, to match with experimental observations [64]. This implied that the local spine and dendritic voltages were on the order of tens of mV for unitary EPSPs as well as during a TBS input (Fig 9*A*), which is consistent with large-amplitude spine and local-dendritic voltages recorded during unitary events [88–91]. We also noted that temporal summation during TBS elicited local dendritic spikes in the immediate vicinity of the synapse (Fig 9*A*), which does not propagate to the soma owing to attenuation during propagation [21].

Consistent with the role of dendritic spines as biochemical compartments, the calcium concentrations which were on the order of 10s of μM at the spine-head dropped significantly with propagation along the spine neck into the dendritic shaft. Specifically, there was almost a 100× attenuation of the calcium levels at the dendritic shaft when compared to that at the spine head (Fig 9*B*). Whereas the high concentrations of calcium at the spine head could be attributed to the high surface to volume ratio (SVR) of the spine compartments, the significant fall in propagating calcium was consequent to the calcium off mechanisms (*i*.*e*., calcium pumps and buffers) expressed in the spine compartments [77,92]. As a consequence of this significant attenuation, the dendritic shaft calcium concentration for a spine-localized synapse was lower than that when the same synapse (with identical receptors and activation dynamics) was located on the dendritic shaft (Fig 9*C*). Despite the large reduction in the dendritic calcium concentration as a consequence of spine localization, the corresponding reduction in pCaMKII was comparatively lower (Fig 9*C*). Overall, despite quantitative differences, the spatiotemporal spread of all molecular species across the signaling topography within the dendritic shaft with a spine-localized synapse (Fig 9*D*) was qualitatively comparable to the signaling spread with a dendrite-localized synapse (Fig 2*G*).

**Fig 10:**
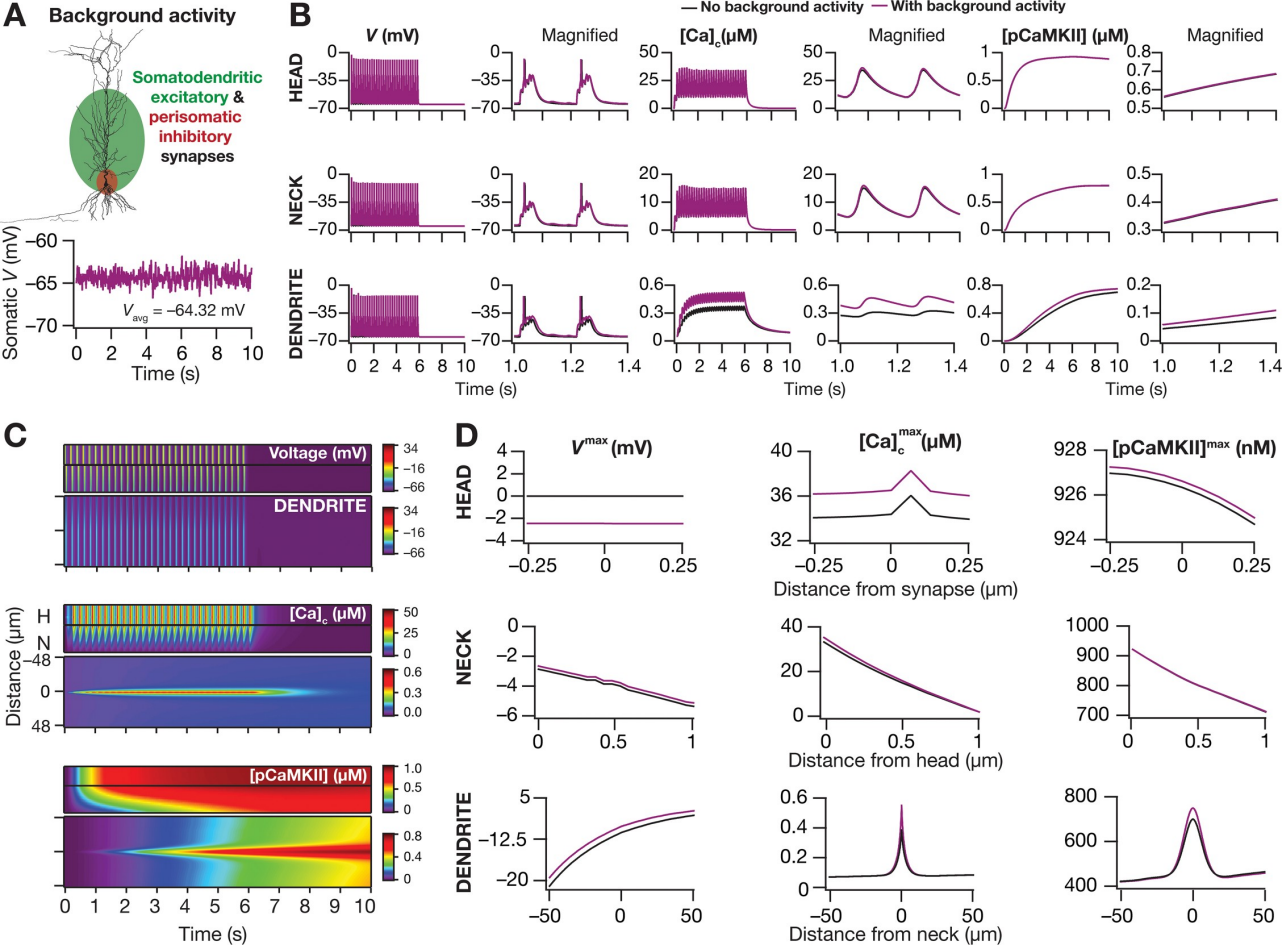
Effect of background synaptic activity on the spatiotemporal spread microdomains. (*A*) *Top*, The morphological reconstruction of the neuron employed for the simulations, with background activity impinging on the somatodendritic arbor. Excitatory inputs impinged on all somatoapical compartments within a 300-μm distance from the soma, and inhibitory synapses made perisomatic contacts. *Bottom*, Stochastic activation of all these synapses resulted in randomized fluctuations in the membrane voltage, with the excitation-inhibition balance maintained to yield an average baseline membrane potential of around –65 mV. (*B*) Voltage (left panels), cytosolic calcium [*Ca*]_c_ (middle) and phosphorylated CaMKII, pCaMKII (right) traces recorded at the center of the spine-head at the synaptic location (top), at the center of spine-neck (middle), at the center of dendritic shaft (bottom) comparing the responses with (purple) or without (black) background synaptic activity during TBS. At the right of each 10 s trace is shown a magnified 400 ms window of two bursts. (*C*) Kymographs showing the spatiotemporal spread of voltage (top), calcium (middle) and pCaMKII (bottom) microdomains at the spine head, neck and dendritic shaft in the presence of background synaptic activity. (*D*) Peak voltage, *V*^max^, maximum value of cytosolic calcium concentration, ${\left[Ca\right]}_{c}^{max}$ and maximum value of phosphorylated CaMKII concentration, [*pCaMKII*]^max^ as recorded at the spine-head (top), spine-neck (middle) and dendritic shaft (bottom) with (purple) or without (black) background synaptic activity along with TBS. All channel conductances were assigned to their baseline values as mentioned in Fig 1. Details of parametric values (of active and passive properties in the oblique dendrite shown in Fig 2*A*) employed for simulations presented in this figure are provided in S1 Table.

Our analyses thus far were performed in the absence of any background synaptic activity. Would the presence of spontaneous background synaptic activity alter the spatiotemporal spread of microdomains across the dendritic structure? To address this, we incorporated randomly activated balanced excitatory and inhibitory synapses throughout the dendritic arbor, resulting in fluctuating membrane potential dynamics (Fig 10*A*). We then compared the dynamics of voltage propagation and the spread of signaling molecules, across the spine and the dendritic shaft, in the presence or absence of the background synaptic activity (Fig 10*B and* 10*D*). Quantitatively, owing to the predominant dendritic presence of background excitatory synapses (Fig 10*A*), there was a small increase in the dendritic response voltage and the consequent calcium influx in the presence of a background voltage fluctuation (Fig 10*B*). Overall, although this small increase in dendritic calcium resulted in a minor enhancement of pCaMKII levels, the spatiotemporal kinetics of the microdomains and the dynamics of their spread were comparable across the spine and the dendrites, irrespective of the presence or the absence of background activity (Fig 10*B*–10*D*).

### Active dendritic regulation of the spatiotemporal spread of signaling microdomains when the synapse was localized on a spine

**Fig 11:**
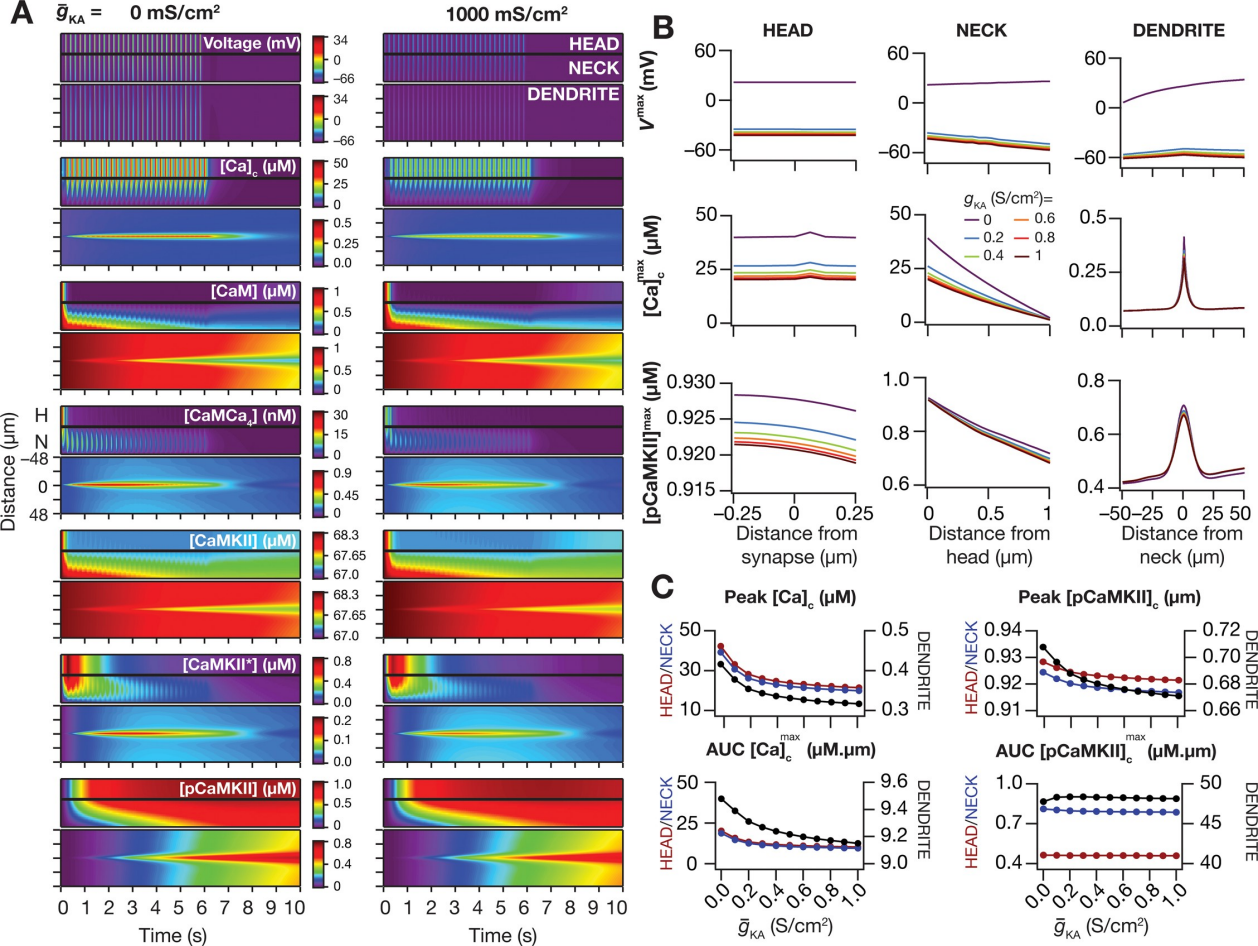
*A-*type potassium channels suppressed the dendritic spread of signaling microdomains in all species of the signaling network, even when the synapse receiving a plasticity-inducing stimulus was localized on a spine. (*A*) Kymographs depicting the spatiotemporal spread of microdomains along the biochemical signaling cascade in response to TBS with the spine/shaft ${\bar{g}}_{KA}$ = 0 mS/cm^2^ (left) and ${\bar{g}}_{KA}$ = 1000 mS/cm^2^ (right). (*B*) Peak voltage, *V*^max^ (top), maximum value of cytosolic calcium concentration, ${\left[Ca\right]}_{c}^{max}$ (middle) and maximum value of phosphorylated CaMKII concentration, [*pCaMKII*]^max^ (bottom) as recorded in response to TBS at the spine-head (left), spine-neck (middle) and dendritic shaft (right) for different spine/shaft ${\bar{g}}_{KA}$ levels. (*C*) Peak value (top panels) and area under the curve, AUC (bottom panels) of ${\left[Ca\right]}_{c}^{max}$ (left) and [*pCaMKII*]^max^ (right) plotted against ${\bar{g}}_{KA}$ for spine head (red), spine-neck (blue) and oblique shaft (black). All channel conductances were assigned to their default values (Fig 1). Details of parametric values (of active and passive properties in the oblique dendrite shown in Fig 2*A*) employed for simulations presented in this figure are provided in S1 Table.

**Fig 12:**
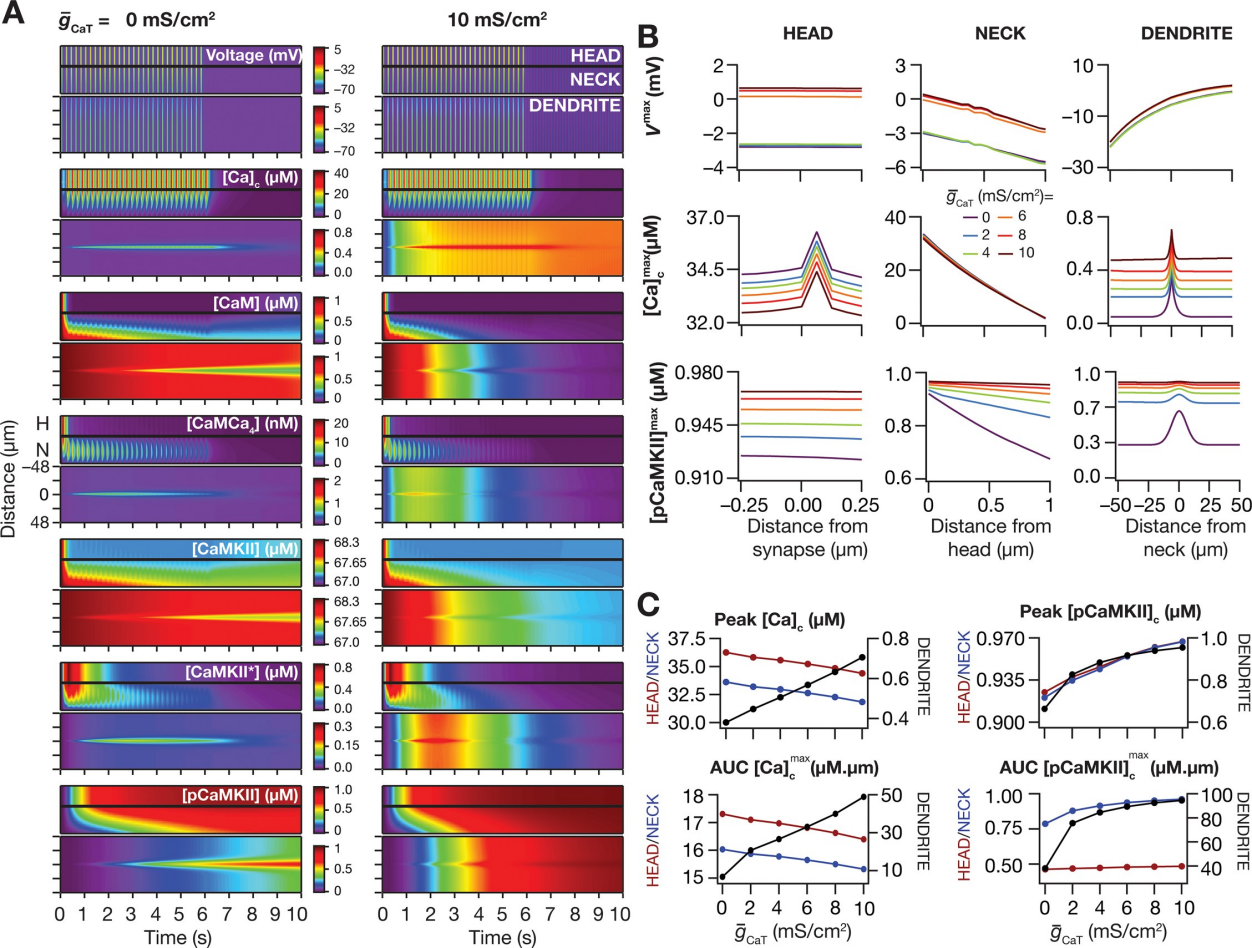
*T-*type calcium channels enhanced the dendritic spread of signaling microdomains in all species of the signaling network, even when the synapse receiving a plasticity-inducing stimulus was localized on a spine. (*A*) Kymographs depicting the spatiotemporal spread of microdomains along the biochemical signaling cascade in response to TBS with the spine/shaft ${\bar{g}}_{CaT}$ = 0 mS/cm^2^ (left) and ${\bar{g}}_{CaT}$ = 10 mS/cm^2^ (right). (*B*) Peak voltage, *V*^max^ (top), maximum value of cytosolic calcium concentration, ${\left[Ca\right]}_{c}^{max}$ (middle) and maximum value of phosphorylated CaMKII concentration, [*pCaMKII*]^max^ (bottom) as recorded in response to TBS at the spine-head (left), spine-neck (middle) and dendritic shaft (right) for different spine/shaft ${\bar{g}}_{CaT}$ levels. (*C*) Peak value (top panels) and area under the curve, AUC (bottom panels) of ${\left[Ca\right]}_{c}^{max}$ (left) and [*pCaMKII*]^max^ (right) plotted against ${\bar{g}}_{CaT}$ for spine head (red), spine-neck (blue) and oblique shaft (black). All channel conductances were assigned to their default values (Fig 1). Details of parametric values (of active and passive properties in the oblique dendrite shown in Fig 2*A*) employed for simulations presented in this figure are provided in S1 Table.

How do *A*-type K^+^ and *T*-type Ca^2+^ channels present on the dendrite regulate microdomain spread when synaptic stimulation arrives on a spine? To address this, we repeated our simulations described in Fig 9 with different densities of *A*-type K^+^ (Fig 11) and *T*-type Ca^2+^ (Fig 12) channels. We found that dendritic *A*-type potassium channels suppressed the spatiotemporal spread of pCaMKII microdomains (Fig 11), whereas dendritic *T*-type calcium channels enhanced the spatiotemporal spread of these downstream microdomains (Fig 12). As noted earlier, mechanistically, the *A*-type K^+^ channels regulate the spread of downstream microdomains by altering the voltage response whereas *T*-type Ca^2+^ channels act through the calcium influx they mediate, without significantly altering the voltage responses. As a consequence of this and because of the tremendous attenuation associated with calcium concentration when the synapse was localized on the spine (Fig 9), the impact of spine localization of the synapse was quantitatively different in the additional presence of these two channel subtypes (Fig 11 *vs*. Fig 12). Specifically, the impact of the additional presence of *A*-type K^+^ channels on pCaMKII spread (Fig 11) was significantly lower compared to that of *T*-type Ca^2+^ channels (Fig 12) when the synapse was localized on the spine, because of the respective indirect *vs*. direct roles of these channels in altering calcium concentration. Together, these results provide further evidence for active dendritic regulation of the spatiotemporal spread of microdomains in plasticity-inducing enzymes through distinct mechanisms and disparate dependencies on synaptic localization profiles.

### Spatiotemporal spread of signaling microdomains in the presence of additional spines in the active dendritic arbor

**Fig 13:**
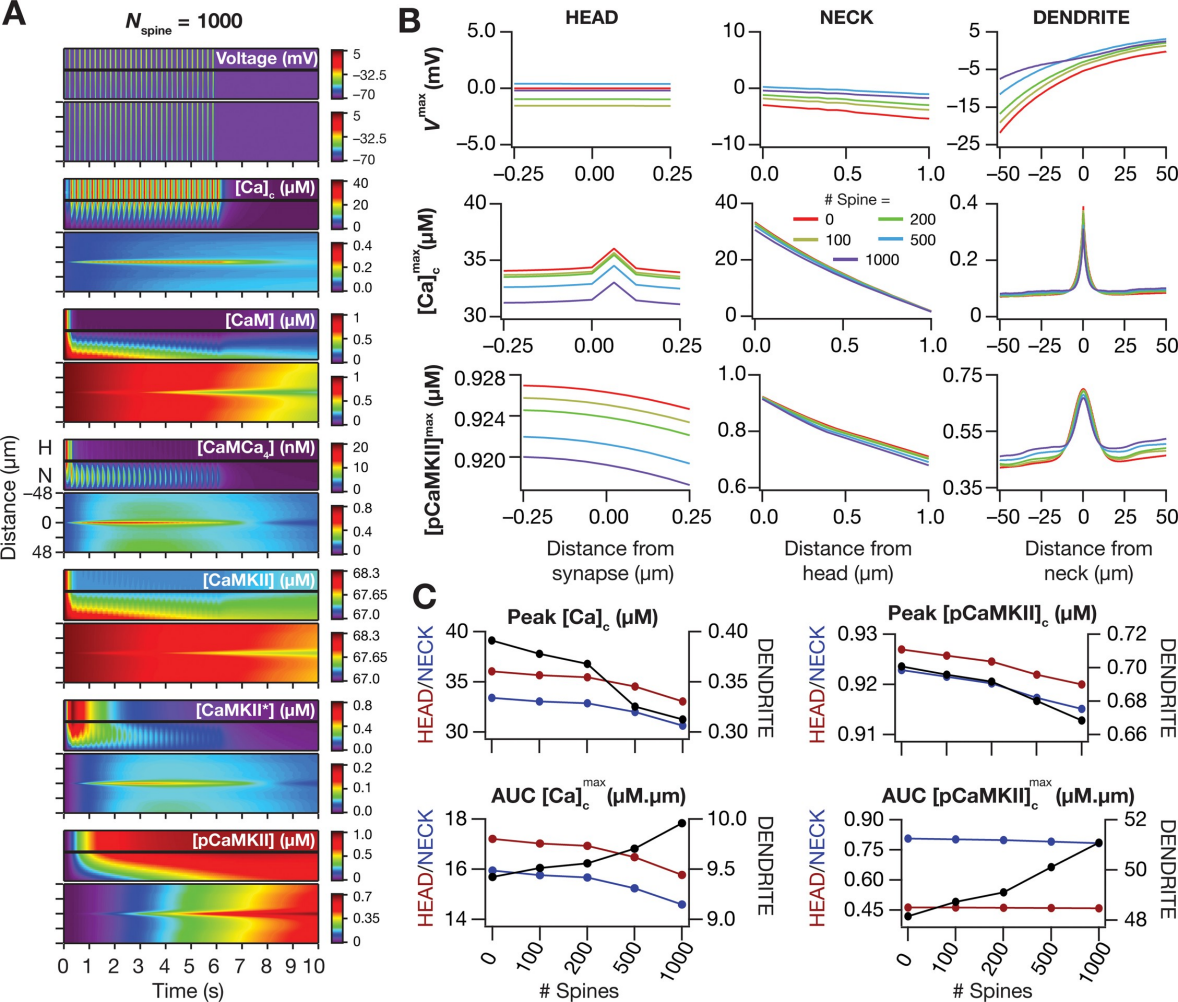
Impact of spine density on the spatiotemporal spread of dendritic signaling microdomains during TBS. *(A)* Kymographs depicting the spatiotemporal spread of microdomains along the biochemical signaling cascade in the presence of 1000 spines incorporated in the chosen dendritic oblique (depicted in Fig 2*A*). (*B*) Peak voltage, *V*^max^ (top), maximum value of cytosolic calcium concentration, ${\left[Ca\right]}_{c}^{max}$ (middle) and maximum value of phosphorylated CaMKII concentration, [*pCaMKII*]^max^ (bottom) as recorded at the spine-head (left), spine-neck (middle) and dendritic shaft (right) for different spine densities. (*C*) Peak value (top panels) and area under the curve, AUC (bottom panels) of ${\left[Ca\right]}_{c}^{max}$ (left) and [*pCaMKII*]^max^ (right) plotted against number of additional spines that were distributed across the dendritic branch. These values are shown for spine head (red), spine-neck (blue) and oblique shaft (black). All head and neck data shown in this figure correspond to the central synapse-containing spine. All channel conductances were assigned to their default values (Fig 1). Details of parametric values (of active and passive properties in the oblique dendrite shown in Fig 2*A*) employed for simulations presented in this figure are provided in S1 Table.

Our analyses thus far were limited to the presence of a single synapse-containing spine that was placed at the center of the oblique dendrite. How would the microdomain spread and the impact of active dendrites on such spread be affected by the presence of other spine structures on the same dendritic structure? To directly address this question, we randomly placed spines spread across the dendritic oblique under consideration in various numbers (100, 200, 500 and 1000). Each of these additional spines had the same passive and active properties, as well as the calcium handling mechanisms, although none of them received any synaptic connections. With one of the distinct configurations with reference to the total number of spines on the oblique dendrite, we stimulated the central synapse-containing spine with the TBS protocol, and compared the spatiotemporal spread of microdomains at various spine densities. As a direct consequence of the overall increase in surface area and the active nature of the membrane, we found that the spatial spread of voltage increased with increase in spine density (Fig 13*A and* 13*B*), especially in the oblique dendrite for propagation towards the trunk. As a consequence of the presence of additional spines, similar to the impact of increased dendritic diameter on microdomain spread [8], we observed a dissipation of calcium and pCamKII microdomains consequent to TBS. Specifically, we noted that the peak values of dendritic [*Ca*] and [*pCaMKII*] resulting from TBS progressively decreased, and the corresponding spatial spread gradually enhanced, with systematic increase in spine density (Fig 13*C*).

**Fig 14:**
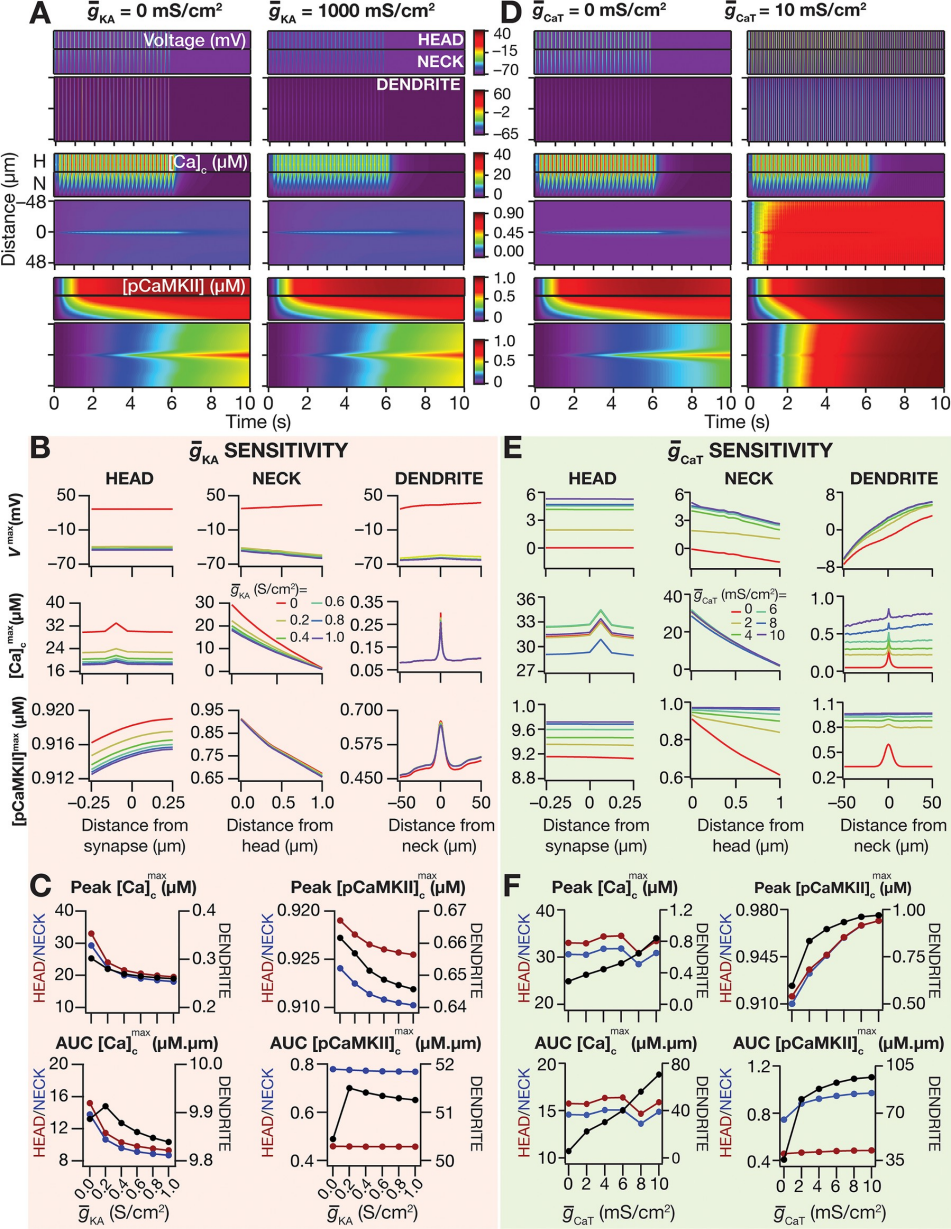
Impact of incorporating dendritic spines on the spatiotemporal spread of signaling microdomains during TBS. (*A*) Kymographs showing the spatiotemporal spread of voltage (top) and calcium (middle) and pCaMKII (bottom) microdomains in the spine head, spine neck and dendritic shaft in the presence of 1000 spines on the chosen dendritic oblique, plotted with ${\bar{g}}_{KA}$ = 0 mS/cm^2^ (left) and ${\bar{g}}_{KA}$ = 1000 mS/cm^2^ (right). (*B*) Peak voltage, *V*^max^, maximum value of cytosolic calcium concentration, ${\left[Ca\right]}_{c}^{max}$ and maximum value of phosphorylated CaMKII concentration, [*pCaMKII*]^max^ as recorded at the spine head (left), spine neck (center) and dendritic shaft (right) for different values of spines/shaft ${\bar{g}}_{KA}$ in the presence of 1000 spines. (*C*) Peak value (top panels) and area under the curve, AUC (bottom panels) of ${\left[Ca\right]}_{c}^{max}$ (left) and [*pCaMKII*]^max^ (right) plotted as a function of spines/shaft ${\bar{g}}_{KA}$ for spine-head (red), spine-neck (blue) and oblique shaft (black). (*D*) Kymographs showing the spatiotemporal spread of voltage (top) and calcium (middle) and pCaMKII (bottom) microdomains in the spine head, spine neck and dendritic shaft in the presence of 1000 spines on the chosen dendritic oblique, plotted with ${\bar{g}}_{CaT}$ = 0 mS/cm^2^ (left) and ${\bar{g}}_{CaT}$ = 10 mS/cm^2^ (right). *(E)* Peak voltage, *V*^max^, maximum value of cytosolic calcium concentration, ${\left[Ca\right]}_{c}^{max}$ and maximum value of phosphorylated CaMKII concentration, [*pCaMKII*]^max^ as recorded at the spine head (left), spine neck (centre) and dendritic shaft (right) for different values of spines/shaft ${\bar{g}}_{CaT}$ in the presence of 1000 spines. *(F)* Peak value (top panels) and area under the curve, AUC (bottom panels) of ${\left[Ca\right]}_{c}^{max}$ (left) and [*pCaMKII*]^max^ (right) plotted as a function of spines/shaft ${\bar{g}}_{CaT}$ for spine head (red), spine-neck (blue) and oblique shaft (black). All head and neck data shown in this figure correspond to the central synapse-containing spine. All channel conductances were assigned to their default values (Fig 1). Details of parametric values (of active and passive properties in the oblique dendrite shown in Fig 2*A*) employed for simulations presented in this figure are provided in S1 Table.

How does the presence of active dendritic components alter microdomain propagation in the presence of spines? Do our conclusions on the role of KA and CaT channels change with the incorporation of dendritic spines? To test this, we repeated our analyses presented in Figs 11 and 12 with 1000 additional spines (~5 spines/μm [93]) randomly distributed on the oblique under consideration (Fig 14). We performed these analyses for distinct densities of KA (Fig 14*A*–14*C*) and CaT (Fig 14*D and* 14*E*) channel densities, with all other parameters (except for the incorporation of spines) remaining the same as the simulations performed to obtain Fig 11 (KA channels) and Fig 12 (CaT channels) respectively. We found our conclusions in the presence of these additional background spines to match with our earlier conclusions, whereby the presence of KA and CaT channels respectively suppressed and enhanced the signaling spread of dendritic calcium and pCaMKII (Fig 14).

### Spatiotemporal spread of signaling microdomains in the presence of paired backpropagating action potentials during theta-burst stimulation

**Fig 15:**
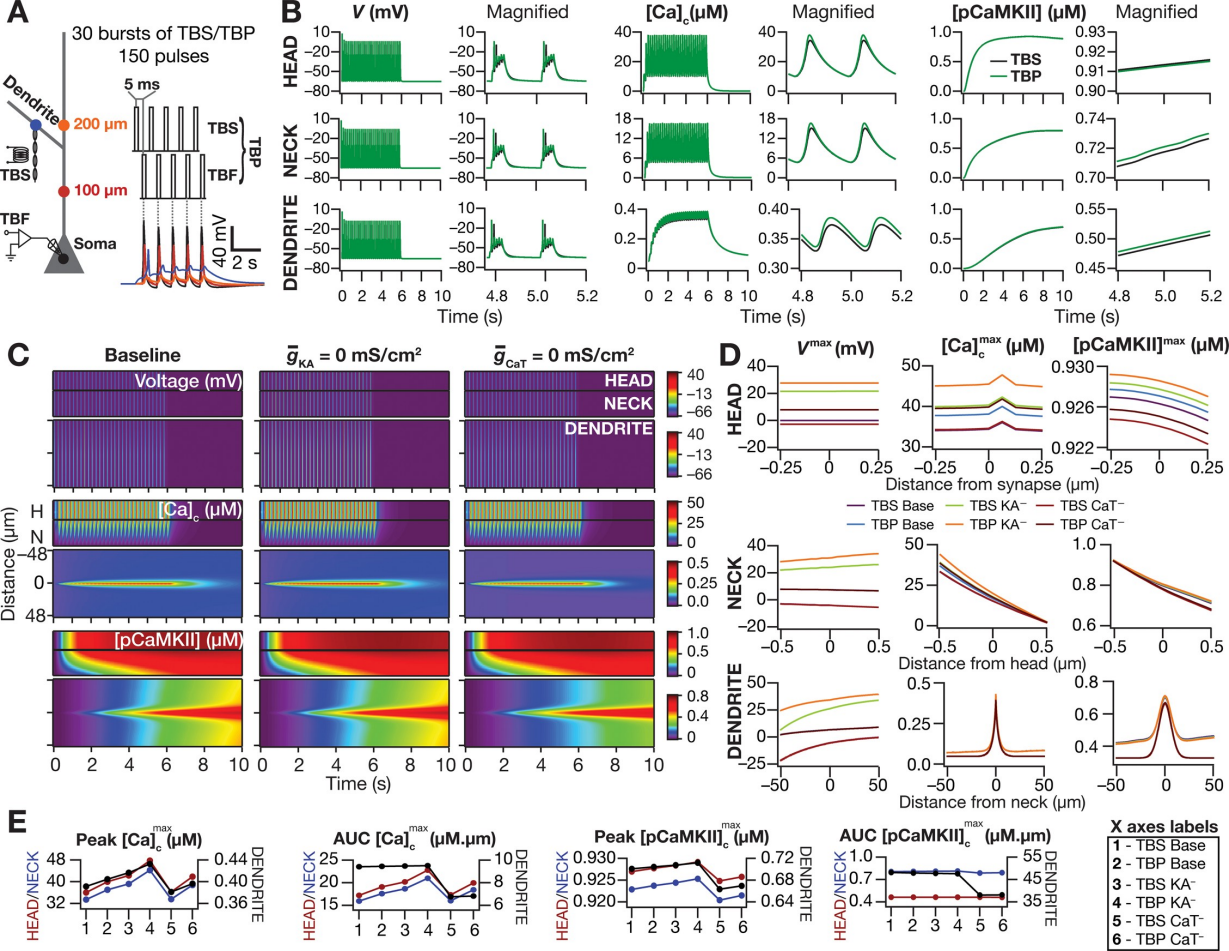
Effect of back propagating action potentials on microdomains. (*A*) *Left*, Schematic of the pyramidal neuron showing the point of synaptic stimulation (for TBS at center of the oblique dendrite, shown in blue) and the point of current injection at the soma (for eliciting theta burst firing, TBF, shown in black), together resulting in the theta burst pairing (TBP) protocol. *Right*, The TBP protocol (1 burst) depicting a 5 ms delay between TBS at the synapse and TBF at the soma. The lower panel shows the corresponding 100 ms time-aligned window of voltage traces recorded from the soma (black), the apical trunk at ~100 μm radial distance (red), at ~200 μm radial distance (orange) and at center of the oblique (blue). (*B*) Voltage (left panels), cytosolic calcium [*Ca*]_c_ (middle) and phosphorylated CaMKII, pCaMKII (right) traces recorded at the center of the spine-head (top), at the center of spine-neck (middle), at the center of dendritic shaft (bottom) comparing responses obtained with TBP (green) and TBS (black) protocol. At the right of each 10 s trace is shown a magnified 400 ms window. (*C*) Kymographs showing the spatiotemporal spread of voltage (top) and calcium (middle) and pCaMKII (bottom) microdomains in the spine head, spine neck and dendritic shaft with the TBP protocol, plotted with baseline levels of ${\bar{g}}_{KA}$ and ${\bar{g}}_{CaT}$ (left), with ${\bar{g}}_{KA}$ knocked out (middle) and ${\bar{g}}_{CaT}$ knocked out (right). (*D*) Peak voltage, *V*^max^, maximum value of cytosolic calcium concentration, ${\left[Ca\right]}_{c}^{max}$ and maximum value of phosphorylated CaMKII concentration, [*pCaMKII*]^max^ as recorded at the spine head (top), spine neck (middle) and dendritic shaft (bottom) with TBS or with TBP for baseline, ${\bar{g}}_{KA}$ knockout and ${\bar{g}}_{CaT}$ knockout cases. (*E*) Peak value (1^st^ and 3^rd^ panel) and area under the curve, AUC (2^nd^ and 4^th^ panels) of ${\left[Ca\right]}_{c}^{max}$ (left two panels) and [*pCaMKII*]^max^ (right two panels) plotted against all the cases shown in *D*, for spine-head (red), spine-neck (blue) and oblique dendritic shaft (black). The legend on the right provides the labels for the *X*-axes of all graphs in this panel. Except for the knockout simulations where specific conductances were set to zero, all channel conductances were assigned to their default values (Fig 1). Details of parametric values (of active and passive properties in the oblique dendrite shown in Fig 2*A*) employed for simulations presented in this figure are provided in S1 Table.

Although TBS is a widely used LTP-induction protocol, a more robust version of the protocol involves pairing some or all synaptic stimulations as part of TBS with somatically initiated action potentials. The version of TBS where all stimulations are paired with appropriately timed action potentials has been referred to as theta-burst pairing (TBP), with the backpropagating action potentials invading a significant proportion of the dendritic tree allowing for enhanced calcium influx during the protocol [12,20,54–58]. Would the presence of paired backpropagating action potentials alter our conclusions in terms of the spread of downstream microdomains and on the impact of active dendritic conductances on such spread? To test this, we paired synaptic stimulations within TBS with backpropagating action potentials (Fig 15*A*), and assessed the impact of such TBP on the spread of downstream microdomains.

As expected from the backpropagation of action potentials, there was a small increase in the voltage responses observed in the oblique dendrite during TBP as compared to responses during TBS (Fig 15*B*). Although the backpropagating action potential amplitude was large on the dendritic trunk (Fig 1*C*), the invasion of backpropagating action potentials into obliques was minimal because of several factors including the branching structure of the dendritic arbor where obliques are made of smaller diameters, the expression of *A*-type K^+^ channels in obliques, and the slow recovery of dendritic sodium channels from inactivation [21,22,36,67,94]. As a consequence of this small increase in voltage response, we found small increases in the calcium and pCaMKII concentrations downstream in the spine and dendritic compartments (Fig 15*B*). These small changes in signaling concentrations also reflected in the spread across the different dendritic segment, overall suggesting that the impact of paired backpropagating action potentials on microdomain spread in oblique dendrites to be minimal (Fig 15*C*–15*E*).

Next, we assessed the specific contributions of the active dendritic conductances in regulating the spread of downstream microdomains with TBS or TBP as the plasticity induction protocol. Specifically, we virtually knocked out (by setting the corresponding conductance to zero, only in the dendritic and spine compartments under consideration) either *A*-type K^+^ or *T*-type Ca^2+^ channels and reassessed the spread of microdomains across the signaling pathway (Fig 15*C*–15*E*). Consistent with our prior observations (Figs 4–8, Figs 11 and 12), we found that knocking out *A*-type K^+^ or *T*-type Ca^2+^ channels respectively enhanced and reduced the peak values and the spread of the calcium and pCaMKII microdomains (Fig 15*C*–15*E*). The small differences between TBP and TBS observed earlier, in terms of TBP eliciting a slightly larger calcium response were reflected in both knockout simulations as well (Fig 15*D and* 15*E*).

Together, our results provide compelling evidence for a critical role for dendritic channels in regulating the spatiotemporal spread of microdomains in plasticity-inducing enzymes, effectuated by changes in excitability and/or calcium influx, in a manner that was invariant to several structural and parametric configurations.

## Discussion

The prime conclusion of this study is that active dendritic conductances play a critical role in regulating the spatiotemporal spread of microdomains associated with plasticity-inducing kinases. We demonstrated this by employing theta-burst synaptic stimulation to a multiscale multicompartmental model that was biochemically and biophysically constrained by experimental measurements. We studied the impact of two inactivating conductances with predominantly dendritic localization profiles, the restorative KA and the regenerative CaT conductances, and showed that they modulate microdomain spread through two distinct mechanisms. Whereas KA channels regulated the spread of pCaMKII microdomains by altering the voltage response to the theta burst stimulus, CaT channels regulated this spread by modulating the calcium influx consequent to TBS without significantly changing the response voltage. Finally, assessing the cross-interactions of KA, CaR and CaT channels (Fig 8) along with their interactions with key structural, biophysical and biochemical parameters (Fig 3, Fig 5, Fig 7, Figs 9–15), we demonstrated the heavy mutual interdependence of several model components in regulating signaling microdomains. Our conclusions unveil a critical role for active dendrites in regulating the spatiotemporal spread of signaling microdomains associated with subcellular molecular networks. Given the physiologically constrained approach that we have employed in this study, our results are also predictions that could be experimentally tested by measuring pCaMKII [43] after TBS or TBP (to different locations along the somatodendritic arbor) in the presence of pharmacological agents to block different channels. Finally, as several cell types express voltage-gated ion channels, and biochemical signaling strength and spread are ubiquitous in their regulatory capacity, our conclusion that voltage-gated ion channels that are present on the plasma membrane could regulate biochemical signaling strength and spread has implications that are not limited only to neurons. In what follows, we discuss the several implications of our conclusions for neuronal physiology and plasticity, and elucidate potential future directions.

### Degeneracy in microdomain spread and its implications for robustness of information transfer in signaling pathways

Our results clearly demonstrate that signaling spread is an active process that is not just governed by cell morphology and the topological motifs and binding kinetics associated with the signaling network [5,8,95–97], but also by the types, kinetics and localization of ion channels in the dendritic arbor. This active amplification (Fig 6, Fig 11, Fig 15) or suppression (Fig 4, Figs 12–15) of biochemical signals and their spread by dendritic ion channel conductances call for a marked rethink of the complexities associated with subcellular signaling networks. Specifically, the numbers associated with ion channel subtypes, their auxiliary subunits, their subcellular localization profiles, and their local or global modulation through neuromodulatory substances or activity-dependent plasticity are staggeringly combinatorial [9,13–15,23]. The additional regulatory capacity of complex dynamics of subcellular signaling by this complex channel network implies a manifold increase in the complexity of molecular signal transduction. These results also clearly demonstrate several disparate combinations of neuronal parameters—associated with morphology, background spine density, signaling motifs, binding kinetics, diffusion and ion channel densities, for instance—could result in similar signal propagation in a given signaling network, pointing directly to degeneracy in spatiotemporal spread of signaling components [98]. In conjunction with recent literature on degeneracy in cellular-scale physiology and plasticity in hippocampal neurons [24,25,39,99–101], these results point to significant degeneracy in hippocampal physiology spanning different scales.

What are the consequences of such combinatorial complexities and degeneracy associated with signaling networks? First, a growing body of established literature that spans several scales of biology has linked complexity and degeneracy as requirements for robustness in biological systems [69,98,102]. In this context, the degeneracy associated with signaling spread could be postulated as a mechanism for achieving robust signaling transfer and spread in the presence of external and internal noise factors. Our demonstration of active suppression or amplification of signaling strength and spread by specific channels provide an additional mechanism by which noise could be selectively suppressed through channels with specific kinetic and voltage-dependence properties. Second, theoretical frameworks at the cellular scale have argued for efficient coding of incoming information [103–105] through ion channel localization and plasticity [14,28,55,106,107], which at the molecular scale has found reflection in terms of maximizing information transfer by matching signaling dynamics to input source statistics [108–111]. The results described here provide a way to unify these two apparently disparate theoretical frameworks (in two different scales) by showing their convergence towards regulation of signal strength and spread. Future studies should endeavor to holistically unify the systems [103–105], cellular [14,28,55,106,107] and molecular [108–110] versions of the efficient coding hypothesis, accounting for input statistics and neuronal response properties at all scales. Third, tunability of information transfer is a critical requirement in several signaling networks [112,113]. With active conductances modulating signaling strength and spread, it is clear that the specific signal that is transmitted would now be dependent on the postsynaptic channel densities (along the pathway of spatial propagation) as well, and not just on the input stimulus and the signaling motifs, thus providing an additional regulatory mechanism for tuning signaling specificity and spread.

### Location-dependence, plasticity and neuromodulation of microdomains and their spread

Given the location-dependent expression profiles of different ion channels, the dependence of signaling spread on active dendritic conductances could directly translate to location-dependence of signaling spread. Specifically, the density of *A*-type K^+^ channels is higher in distal dendrites implying a significant suppression of the spread of signaling at distal locations. However, as different channel conductances have very different channel localization profiles and neuronal physiology is an emergent outcome of intricate and complex spatial and kinetic interactions between these different channels [9,13–15,23,39,99,114], the spread of downstream signaling molecules would also be determined by these interactions (Fig 3, Fig 5, Figs 7–15). Further, as several of these channels have non-uniform distributions, our results imply that the spread of downstream signaling microdomains might not be symmetric with reference to the synaptic location (the point of origin of the second messenger). Such a scenario provides a putative mechanism for spatiotemporally steering the spread and specificity of downstream signaling by regulating ion channel properties and localization profiles. Finally, given that oblique dendritic branches could have different branch strengths as a consequence of differences in *A-*type K^+^ channel expression [22], our results present a testable prediction that CaMKII-dependent plasticity could spread to channels and receptors located over larger distances in branches with lower *A-*type K^+^ channel expression (oblique dendrites with higher branch strength). In branches where the *A-*type K^+^ channel density is higher, on the other hand, could have the plasticity confined to a much smaller region owing to the constricted spread of the CaMKII microdomains. In this context, systematic analyses of the extent of spatiotemporal *influence* of different ion channel clusters on signaling microdomains, and of the dependence of such *influence fields* on the inhomogeneous distribution of different ion channels, the morphology of the dendritic arbor, the direction of propagation of voltage signals, the presence of background synaptic activity and the specific location of the channel in the dendritic arbor (*e*.*g*., on the trunk *vs*. on the thinner obliques) would provide further quantitative insights into the roles of active dendrites on the spread of microdomains [114].

Our study presents the possibility of location-dependent expression profiles of channels and their impact on voltage and calcium signals as potential mechanisms to steer downstream signaling molecules. However, quantitative links between voltage recordings, calcium transients and the spatiotemporal spread of downstream microdomains should not be generalized without specific analyses of the channels and the signaling components expressed in a specific system. First, although voltage transients provide one trigger for cytosolic calcium influx, they are not the only source of calcium, with the ER and other store-operated mechanisms playing a role in regulating calcium influx [26,115–120]. Second, the calcium transients (both amplitude and spread) are critically regulated by several factors (Figs 3–14) including surface-area-to-volume ratio, spine densities in specific dendritic arbors, the densities of channels, receptors, several pumps, transporters and buffer concentrations [26,77,79,81,87,92,121]. Therefore, factors such as altered surface-area-to-volume ratio and nonhomogeneous distribution of any of these components would critically affect calcium amplitude and spread, and alter calcium transients and downstream signaling *independent* of changes in voltage transients [100]. Third, the signaling spread of downstream molecular species is not a simple function of voltage and calcium transients, but is also critically dependent on several factors including surface-area-to-volume ratio of the compartment, background spine densities, the binding affinities, diffusion and subcellular localization of the different signaling components, the topology of the signaling cascade and on the presence of negative regulators upstream [2,4,8,11]. Finally, all these components—the ones that govern the voltage and calcium transients and those that govern the downstream signaling—critically interact with each other through several routes (Fig 3, Fig 5, Figs 7–15), implying a complex parametric and interactional landscape created by the presence of active dendritic components in regulating signaling microdomains.

Based on these observations, we also postulate that active dendrites constitute a putative mechanism to regulate clustered plasticity, a phenomenon where spatially adjacent synapses undergo concurrent plasticity, in dendritic branches [122–127]. Under such a postulate, activity-dependent plasticity [13–15,19,20,22] and/or state-dependent neuromodulation [65,72,128,129] of active dendritic conductances could control the degree of compartmentalization of experience-dependent synaptic plasticity on specific dendrites, thereby regulating the degree of clustering of functional synaptic inputs [123,124,126,127]. Active dendritic conductances, especially restorative conductances, are therefore very critical in confining the spatiotemporal spread of plasticity, thereby assigning a dendritic branch as a fundamental functional unit of biophysical and biochemical signal integration [122–127]. Finally, such regulation of signaling spread by active dendrites, coupled with well-established plasticity and modulation in these dendritic conductances [13–15,19,20,22,65,72,128,129] imply that signaling spread in any signaling molecule in a dendritic branch is dynamic and state-dependent, and that it would be inappropriate to assign a static picture for such a complex dynamical system. This dynamical spread in signaling microdomains has to be assessed accounting for morphological properties of the structure, the network topology, the binding and diffusion kinetics of each signaling component, active dendritic conductances and their properties, the different substrates for the plasticity-inducing enzymes, the localization of all these components, and importantly behavioral state- and activity-dependent modulation and/or plasticity in each of these components.

### Limitations of the analyses and future directions

In accommodating the significant computational complexity associated with a reaction-diffusion system with stringent requirements on spatial discretization into a morphologically realistic model, we restricted our attention in this study to only a few channel types that are expressed in hippocampal dendrites. Although our analyses provide compelling evidence for a critical role for dendritic ion channels in regulating signaling spread, future studies should systematically characterize the impact of these channel types, including the HCN, calcium-activated potassium and inwardly rectifying potassium channels, on different signaling pathways. Another limitation of our analyses is the absence of metabotropic receptors and calcium-induced calcium release (CICR) and mechanisms associated with the activation of these metabotropic receptors. Specifically, it is established that CICR and other store-related mechanisms significantly interact with plasma membrane ion channels in yielding a complex landscape for the passive and active propagation of calcium within the cytosol [26,115–120]. These CICR mechanisms are critically tied to the activation of specific metabotropic receptors, and have been shown to be involved in certain forms of plasticity through specific signaling cascades [130,131]. Future studies should incorporate these ER-related mechanisms, metabotropic receptors and the complex interactions between dendritic ion channels and ER mechanisms [26,132] in deriving more routes for active-dendritic regulation of signaling microdomains through such interactions.

Although our choice of the signaling pathway was motivated by its physiological relevance to plasticity induction and spread, and by the requirement to reduce computational complexity, the pathway is significantly oversimplified from the standpoint of known complexities in signaling motifs and pathways [4,95–97]. Given the possibility that different ion channels and their spatial and kinetic interactions [39,99,114] could differentially interact with different biochemical network motifs in different morphological structures [7,8], future studies should assess such multiscale interactions towards robustness to internal/external noise, increases in information transmission and storage capacity, efficient encoding of afferent signals and towards tunability of signaling specificity and spread [14,28,39,55,69,98,99,102–114]. From a generic standpoint, the basic conclusion of our analyses on a critical role for voltage-gated ion channels and their structural/functional interactions with the different signaling components in regulating signaling microdomains is extendible to other neuronal structures expressing active dendrites, and even to other cell types expressing voltage-gated ion channels. However, given the critical dependencies on these conclusions on the specific channels and their distributions, on cellular morphologies and on the topology of the signaling cascade, future studies should build cell-specific multiscale models that expand on the analyses presented here. Finally, our reaction-diffusion model is a deterministic system that coupled compartmental modeling with partial differential equations (of continuous concentrations of signaling species) to assess the spread of microdomains. Although such continuous deterministic models have provided significant insights into biological signal transduction, a more realistic approach to the problem would be to employ a stochastic discrete system (involving number of molecules of signaling species) that would mimic the stochastic interactions of individual molecules within biological systems [4,5,11,133–135].

## Methods

We employed a multiscale, multicompartmental, morphologically realistic, conductance-based model that accounted for the biophysics of electrical signaling and the biochemistry of calcium handling and downstream enzymatic signaling in a hippocampal neuron. Parameters associated with these were derived from electrophysiological and biochemical data from hippocampal pyramidal neurons. The biophysical model for electrical signaling and the models for calcium on and off mechanisms, including diffusion, were adapted from previous literature [25,26,38–40]. The signaling pathway, and the biochemical models for enzymatic signaling downstream of calcium were adopted from [10,41–52,97,136–138].

### Spatial discretization and passive properties

A morphologically realistic multicompartmental 3D model (Fig 1*A*) was constructed from a reconstructed CA1 pyramidal neuron morphology (*n123*) taken from the Neuromorpho database [139,140]. Passive parameters were set as follows: *C*_m_ = 1 μF/cm^2^; *R*_*m*_ and *R*_*a*_ for various compartments along the somato-apical trunk were functions of radial distance of the compartment from the soma, *x* [55]:

$$R_{a}(x)=R_{a}^{som}+\frac{\left(R_{a}^{end}-R_{a}^{som}\right)}{1+exp\left(\frac{300-x}{50}\right)}\Omega .cm$$ (1)

$$R_{m}(x)=R_{m}^{som}+\frac{\left(R_{m}^{end}-R_{m}^{som}\right)}{1+\exp\left(\frac{300-x}{50}\right)}k\Omega .cm^{2}$$ (2)

where $R_{m}^{som}$ = 125 kΩ.cm^2^ and $R_{a}^{som}$ = 120 Ω.cm were values at the soma, and $R_{m}^{end}$ = 85 kΩ.cm^2^ and $R_{a}^{end}$ = 70 Ω.cm were values assigned to the terminal end of the apical trunk (which was ~425 μm away from the soma for the reconstruction under consideration). The non-uniformity of passive properties considered here follows from evidence from the literature that has argued for the necessity of such non-uniformity to match electrophysiological measurements [55,114,118,141–143], and has been specifically employed to match passive input resistance of the somato-apical trunk [39]. The basal dendrites, the axonal compartments, and apical obliques had somatic *R*_m_ ($R_{m}^{som}$ = 125 kΩ.cm^2^) and *R*_a_ ($R_{a}^{som}$ = 120 Ω.cm) [39]. Except for the oblique where the signaling microdomains were assessed (Fig 2*A*) this neuronal model was compartmentalized using the *d*_λ_ rule [144] to ensure that each compartment was smaller than 0.1 λ_100_, where λ_100_ is the space constant, computed at 100 Hz for the section under consideration. This process resulted in 873 compartments for the entire neuronal structure. As eliminating numerical errors in the estimation of Ca^2+^ signals (whose space constant is on the order of 0.5 μm) requires much smaller compartment sizes than such electrical compartmentalization [26,63,145], the oblique (of total length 193 μm) highlighted in Fig 2*A* was compartmentalized to 2000 compartments (making each compartment size to be around 97 nm). This spatial discretization procedure resulted in a total number of 2864 compartments in the neuronal structure.

### Active conductances across the somatodendritic arbor

Six different types of voltage gated ion channels (VGIC) were incorporated into these models: a fast Na^+^ (NaF), a delayed rectifier K^+^ (KDR), a hyperpolarization-activated cyclic-nucleotide-gated non-specific cationic (HCN), an *A*-type K^+^ (KA) and *R*- (CaR) and *T*-type Ca^2+^ (CaT). Biophysically realistic, Hodgkin-Huxley type conductance-based models derived from hippocampal pyramidal neurons were employed for modeling all these channels. The kinetics, voltage-dependencies and subcellular localization profiles of these channels were derived from hippocampal pyramidal neurons, and the details are provided below and in S1 Text.

#### Sodium and potassium channels

The densities of NaF and KDR channels were uniform across the somatodendritic arbor [16,18,37]. To account for the lower membrane potential threshold for spike generation at the axon initiation segment, the Na^+^ channel density at this location was increased five fold compared to the somato-dendritic values [146]. The rest of the axon was considered as passive. To account for slower recovery from inactivation of dendritic Na^+^ channels, an extra inactivation gate was added while modeling the channel kinetics for dendritic Na^+^ channels [36,37]. The Na^+^ channel density in apical and basal dendrites was the same as the soma. ${\bar{g}}_{KDR}$ and ${\bar{g}}_{Na}$ were fixed at 10 mS/cm^2^ and 16 mS/cm^2^ respectively. These parameters were adjusted so as to obtain unattenuated action potential backpropagation across the somatoapical trunk [16,37].

In incorporating KA channels, kinetics and voltage-dependencies of the channels were set to be different for the proximal (≤100 μm from the soma) and distal (>100 μm) compartments [16,37]. The density of KA channels was linearly increased as a function of distance of the compartment from the soma [16,37]. The density distribution of KA channels in the apical dendritic locations was (Fig 1*B*):

$${\bar{g}}_{KA}(x)=g_{KA}^{som}\left(1+\frac{8x}{100}\right)\;{\mathrm{mS}/\mathrm{cm}}^{2}$$ (3)

where ${\bar{g}}_{KA}(x)$ represented the maximal KA conductance at radial distance *x* μm from the soma, and $g_{KA}^{som}$ represented the maximal conductance value for KA channel at the soma, with a default value of 3.1 mS/cm^2^ (translating to 3.1–108.25 mS/cm^2^ over a radial span of 425 μm; Fig 1*B*). This gradient was set such that the backpropagating action potential amplitude at a trunk location ~300 μm from the soma was around 20 mV [16,37,147]. Reversal potentials for Na^+^ and K^+^ channels were set at 55 and –90 mV, respectively.

#### HCN channels

The kinetics and voltage-dependence of the current through HCN channels were derived from hippocampal pyramidal neuron recordings, with their reversal potential set at –30 mV [17,148,149]. HCN-channel density [17,55,150] along the somatodendritic arbor was set as (Fig 1*B*):

$${\bar{g}}_{h}(x)=g_{h}^{som}\left(1+\frac{12}{1+exp((320-x)/50)}\right)\;\mu {S/\mathrm{cm}}^{2}.$$ (4)

This gradient in HCN channel density and the associated passive properties ensured that *R*_in_ decreased from ~65 MΩ at the soma to ~40 MΩ at a trunk location 300 μm away from the soma (Fig 1*D*). $g_{h}^{som}$, the maximal value of the somatic HCN conductance density was set at 25 μS/cm^2^ (translating to 25–291.68 μS/cm^2^ over a radial span of 425 μm; Fig 1*B*). The half-maximal activation voltage was set to –82 mV for compartments <100 μm away from the soma and hyperpolarized linearly up to –90 mV for compartments up to 300 μm away from the soma, beyond which it stayed at –90 mV [17,55]. For basal dendrites, HCN-channel density and kinetics were kept the same as those in the somatic compartments, and axonal compartments lacked HCN channels.

#### Calcium channels

The density of CaT channels increased as a function of distance from the soma, with their kinetics and voltage-dependence derived from hippocampal pyramidal neurons [18,99,151,152].

$${\bar{g}}_{CaT}(x)=g_{CaT}^{som}\left(1+\frac{30}{1+exp((350-x)/50)}\right)\;\mu {S/\mathrm{cm}}^{2}$$ (5)

where $g_{CaT}^{som}$ represented the density of CaT conductances at the soma, with a default value of 80 μS/cm^2^ (translating to 0.08–2.03 mS/cm^2^ over a radial span of 425 μm; Fig 1*B*). In the basal dendrites, CaT channel density was same as that of the soma. CaR channels, when introduced (Figs 4 and 5, Figs 7 and 8), were specific only to the oblique under consideration (Fig 2*A*), with channel kinetics and voltage dependence derived from hippocampal pyramidal neuron recordings [18,153]. In the baseline model, the CaR channels were absent across the somatodendritic arbor, and were inserted into the oblique under consideration only to assess the sensitivity of our conclusions to the presence of CaR channels (Figs 4 and 5, Figs 7 and 8). The specific value of *g*_CaR_ is mentioned in the figure or their legends when it was non-zero. It may be noted that there are several lines of evidence to show that both *T*- and *R*-type calcium channels are expressed at higher densities in the dendrites and spines of CA1 pyramidal neurons. First, direct cell-attached recordings from CA1 pyramidal neuron dendrites have shown the high-density expression of these channels in dendritic structures [18]. Second, there are lines of evidence emanating from calcium imaging experiments that point to the expression of *R*-type calcium channels (Ca_v_2.3) in spines [154,155], and the presence of dendritic *T*-type calcium channels [71]. Finally, there are also lines of evidence that implicate *T*-type [73,74] and *R*-type [74–76] channels as a local calcium source in the induction of plasticity or as a local target of activity-dependent plasticity. The CaT and CaR currents were modeled using the Goldman-Hodgkin-Katz, GHK formulation [156,157] with the default values of external and internal calcium concentrations set at 2 mM and 50 nM, respectively [25,152]. Specifically, the calcium currents through CaT and CaR channels were specified using the following equation [158]:

$$I_{Ca}^{}(v,t)={\bar{g}}_{Ca}\;S_{Ca}(t)\;v\left(\frac{1-\frac{{\left[Ca\right]}_{i}}{{\left[Ca\right]}_{o}}exp\left(\frac{2vF}{RT}\right)}{1-exp\left(\frac{2vF}{RT}\right)}\right)$$ (6)

where *S*_*Ca*_ (*t*) represented the product of the activation and inactivation state variables for the respective channel (see S1 Text), ${\bar{g}}_{Ca}$ specified the maximal conductance density of the channel (S/cm^2^), with [*Ca*]_*i*_ = 50 nM and [*Ca*]_*o*_ = 2 mM.

### Glutamate receptors and synaptic stimulation protocol

A canonical glutamate synapse consisting of colocalized NMDARs and AMPARs was placed at the mid point of a proximal oblique represented in Fig 2*A*. Specifically, the NMDAR current was modeled as a combination of three different types of ionic currents namely Ca^2+^, Na^+^ and K^+^ [29]:

$$I_{NMDA}(v,t)=I_{NMDA}^{Na}(v,t)+I_{NMDA}^{K}(v,t)+I_{NMDA}^{Ca}(v,t)$$ (7)

where,

$$I_{NMDA}^{Na}(v,t)={\bar{P}}_{NMDA}\;P_{Na}\;s(t)\;MgB(v)\frac{vF^{2}}{RT}\left(\frac{{\left[Na\right]}_{i}-{\left[Na\right]}_{o}\exp\left(-\frac{vF}{RT}\right)}{1-\exp\left(-\frac{vF}{RT}\right)}\right)$$ (8)

$$I_{NMDA}^{K}(v,t)={\bar{P}}_{NMDA}\;P_{K}\;s(t)\;MgB(v)\frac{vF^{2}}{RT}\left(\frac{{\left[K\right]}_{i}-{\left[K\right]}_{o}\exp\left(-\frac{vF}{RT}\right)}{1-\exp\left(-\frac{vF}{RT}\right)}\right)$$ (9)

$$I_{NMDA}^{Ca}(v,t)={\bar{P}}_{NMDA}\;P_{Ca}\;s(t)\;MgB(v)\frac{4vF^{2}}{RT}\left(\frac{{\left[Ca\right]}_{i}-{\left[Ca\right]}_{o}\exp\left(-\frac{2vF}{RT}\right)}{1-\exp\left(-\frac{2vF}{RT}\right)}\right)$$ (10)

where ${\bar{P}}_{NMDA}$ is the maximum permeability of the NMDAR; *P*_*Ca*_ = 10.6, *P*_*Na*_ = 1, *P*_*K*_ = 1 [159,160]. Default values of concentrations were (in mM): [*Na*]_*i*_ = 18, [*Na*]_*o*_ = 140, [*K*]_*i*_ = 140, [*K*]_*o*_ = 5, [*Ca*]_*i*_ = 50 × 10^−6^, [*Ca*]_*o*_ = 2. The concentrations for sodium set its equilibrium potential at +55 mV and that for potassium at –90 mV. *MgB(v)* governs the magnesium dependence of the NMDAR current, given as [161]:

$$MgB(v)={\left(1+\frac{{\left[Mg\right]}_{o}\exp(-0.062v)}{3.57}\right)}^{-1}$$ (11)

with the default value of [*Mg*]_*o*_ set at 2 mM. *s*(*t*) governed the kinetics of the NMDAR current, and was set as:

$$s(t)=a\left(\exp\left(-\frac{t}{\tau_{d}}\right)-\exp\left(-\frac{t}{\tau_{r}}\right)\right)$$ (12)

where *a* is a normalization constant, making sure that 0 ≤ *s*(*t*) ≤ 1,τ_*d*_ is the decay time constant, τ_*r*_ is rise time, with τ_*r*_ = 5 ms, and τ_*d*_ = 50 ms.

Current through the AMPAR was modeled as the sum of currents carried by sodium and potassium ions:

$$I_{AMPA}(v,t)=I_{AMPA}^{Na}(v,t)+I_{AMPA}^{K}(v,t)$$ (13)

where,

$$I_{AMPA}^{Na}(v,t)={\bar{P}}_{AMPA}\;P_{Na}\;s(t)\frac{vF^{2}}{RT}\left(\frac{{\left[Na\right]}_{i}-{\left[Na\right]}_{o}exp\left(-\frac{vF}{RT}\right)}{1-exp\left(-\frac{vF}{RT}\right)}\right)$$ (14)

$$I_{AMPA}^{K}(v,t)={\bar{P}}_{AMPA}\;P_{K}\;s(t)\frac{vF^{2}}{RT}\left(\frac{{\left[K\right]}_{i}-{\left[K\right]}_{o}exp\left(-\frac{vF}{RT}\right)}{1-exp\left(-\frac{vF}{RT}\right)}\right)$$ (15)

where ${\bar{P}}_{AMPA}$ is the maximum permeability of the AMPAR, whose default value was set at 20 nm/s, yielding a unitary voltage response of around 0.3 mV. *P*_*Na*_ was taken to be equal to *P*_*K*_ [162]. *s*(*t*) was the same as that for the NMDA receptor, but with τ_r_ = 2 ms and τ_d_ = 10 ms [29]. ${\bar{P}}_{NMDA}=NAR\times {\bar{P}}_{AMPA}$, with a default value for the NMDAR:AMPAR ratio (NAR) set at 1.5.

We employed theta burst stimulation (TBS), an established protocol for induction of synaptic plasticity [53], to assess the spread of signaling microdomains in the model. For TBS, the synapse was stimulated with a burst of 5 action potentials at 100 Hz, and this burst was repeated 30 times at 200 ms interval (5 Hz; theta frequency) each (Fig 2*A*). To analyze the effect of back-propagating action potentials (bAPs) initiated during the induction protocol on the spatio-temporal dynamics of microdomains, we used the theta burst pairing (TBP) protocol that has been employed for inducing neuronal plasticity [20,54,55]. In this protocol, each synaptic stimulation pulse during TBS was followed by a current pulse injection at the soma (current clamp amplitude = 2.5 nA; duration of each pulse = 1 ms, each of which initiates an axo-somatic action potential that backpropagates into the dendritic arbor as well), with a time lag of 5 ms (Fig 15*A*). This led to the TBS to be paired with theta-burst firing (TBF), together resulting in the TBP protocol [20,54,55].

### Calcium dynamics

The overall Ca^2+^ dynamics was modeled as a function of various mechanisms that lead to changes in cytosolic Ca^2+^, [*Ca*]_c_, within a neuron. The partial differential equation governing the cytosolic Ca^2+^ dynamics was [26,38,163,164]:

$$\frac{\partial [Ca]}{\partial t}=D_{ca}\nabla^{2}[Ca]+\beta \left(J_{leak}-J_{SERCA}\right)+R_{buf}+J_{CC}-J_{pump}$$ (16)

where *D*_Ca_ represents experimentally determined diffusion coefficient for Ca^2+^ [165,166] and β constitutes the density of SERCA pumps and leak channels on the ER along the somato-dendritic axis. *J*_*CC*_, *J*_*SERCA*_, *R*_*buf*_, *J*_*pump*_ and *J*_*leak*_ represent the Ca^2+^ flux due to calcium channels, SERCA pumps, buffering, plasma membrane Ca^2+^ extrusion pumps and the ER leak channels, respectively. Radial diffusion of Ca^2+^ was incorporated by radial compartmentalization of the neuronal compartments into 4 concentric annuli, and diffusion along longitudinal axis of the neuron was also implemented [144]. The concentrations of individual molecular species (*e*.*g*., calcium, calmodulin) are reported for the outermost annulus of different longitudinal compartments. Detailed descriptions for each of the fluxes are presented below, and a list of all parameters employed for modeling calcium dynamics, with values derived from previous studies [26,38,163,164], are listed in Table 1.

**Table 1:** Parameters employed in modeling calcium dynamics.

Parameter	Symbol	Value	Unit
Average amplitude of SERCA pump uptake	*V*_max_	10^−4^	mM ms^–1^
Dissociation constant for Ca^2+^ binding to a pump	*K*_P_	0.27	μM
Initial cytosolic Ca^2+^ concentration	[*Ca*]_0_	0.05	μM
Ca^2+^ concentration in ER	[*Ca*]_ER_	400	μM
Average rate of Ca^2+^ flux density at the plasma membrane	γ	8.0	μm s^–1^
Threshold condition for Ca^2+^ extrusion at plasma membrane	[*Ca*]_crt_	0.2	μM
Total static buffer concentration	[*B*_s_]	450	μM
Static buffer dissociation constant	*K*_s_	10	μM
Ca^2+^ diffusion coefficient	*D*_Ca_	220	μm^2^s^–1^

*ER leak channels and SERCA pump*. The rate of Ca^2+^ influx into the cytoplasm through ER leak channels was modeled as [163]:

$$J_{leak}=L\left(1-\frac{\left[Ca\right]}{{\left[Ca\right]}_{ER}}\right)\mathrm{mM}/\mathrm{ms}$$ (17)

where *L* was chosen such that there was no net flux of Ca^2+^ between the ER and the cytosol at resting state.

The Ca^2+^ uptake by sarcoplasmic endoplasmic reticulum Ca^2+^ ATPase (SERCA) pump was modeled as [163]:

$$J_{SERCA}=V_{max}\frac{{\left[Ca\right]}^{2}}{{\left[Ca\right]}^{2}+K_{P}^{2}}\mathrm{mM}/\mathrm{ms}$$ (18)

where *V*_max_ depicts the maximal rate of pump and *K*_*P*_ is dissociation constant of Ca^2+^ binding to the pump.

#### Calcium buffering

We incorporated buffers uniformly through the somatodendritic axis. *R*_*buf*_ defined the rate of change in [*Ca*]_c_ due to buffers:

$$R_{buf}=-k_{on}[Ca][B_{s}]+k_{off}[CaB_{s}]$$ (19)

where [*B*_s_] defined the concentration of stationary buffers, [*Ca B*_s_] represented the concentration of Ca^2+^ bound to stationary buffer. Bound buffer was considered to be in rapid equilibrium with the surrounding Ca^2+^ and a pseudo-steady-state approximation was applied to simulate buffering [26,38,163,165]:

$$\frac{d[B_{s}]}{dt}=-\frac{d[CaB_{s}]}{dt}=R_{buf}$$ (20)

$$K_{s}=\frac{k_{s}^{off}}{k_{s}^{on}},$$ (21)

where $k_{s}^{on}$ (= 1000 /mM-ms) and $k_{s}^{off}$ were the on- and off-rate constants for Ca^2+^ binding to the buffer, and *K*_s_ (= 10 μM) represented the buffer dissociation constant.

#### Voltage- and ligand-gated calcium channels

Ca^2+^ current through VGCCs (*R*- and *T*-type) and through NMDARs was converted to Ca^2+^ concentration as [167]:

$$J_{CC}=\frac{I_{Ca}\times \pi \times diam}{300\times F}\mathrm{mM}/\mathrm{ms}$$ (22)

where *I*_Ca_ depicts the net inward Ca^2+^ current, *diam* stands for the diameter of the compartment, and *F* is Faraday’s constant.

#### Plasma membrane calcium extrusion pump

Ca^2+^ extrusion through plasma membrane pumps was regulated by a threshold on the [*Ca*]_c_. The pumps were inactive below a critical Ca^2+^ concentration, [*Ca*]_crt_, above which the extrusion rate depended linearly on [*Ca*]_c_ [163]:

$$J_{pump}=\{\begin{array}{ccc} \gamma \left({\left[Ca\right]}_{c}-{\left[Ca\right]}_{crt}\right) & : & {\left[Ca\right]}_{c}\geq {\left[Ca\right]}_{crt}\\ 0 & : & \mathrm{otherwise} \end{array}$$ (23)

where [*Ca*]_*crt*_ was set at 0.2 μM based on previous experimental observations [168], and γ defines the sensitivity of pump extrusion [163].

### Signaling pathway and associated reaction-diffusion processes

The signaling pathway that was assessed in this study is pictorially represented in Fig 1*E* [41–52]. The specific binding reactions are listed below, with all associated parameters tabulated as Table 2 [10,97,136–138]. First, we modeled the binding of cytosolic calcium (*Ca*) to calmodulin (*CaM*) to form the calcium-calmodulin complex (*CaMCa*4) as:

**Table 2:** Parameters employed in defining kinetics and diffusion of downstream signaling components.

Parameter	Symbol	Value	Unit
Total calmodulin concentration	[*CaM*]_*T*_	1	μM
Diffusion constant for calmodulin	*D*_CaM_	4	μm^2^ s^–1^
Forward reaction rate for calcium-calmodulin binding	$k_{CaM}^{on}$	8.4848	mM^–1^ ms^–1^
Dissociation constant for calcium-calmodulin binding	*K*_*CaM*_	1.0001	μM
Total CaMKII concentration	[*CaMKII*]_*T*_	70	μM
Diffusion constant for CaMKII	*D*_CaMKII_	1.6	μm^2^ s^–1^
Forward reaction rate for CaMCa4-CaMKII binding	$k_{CaMKII}^{on}$	100.004	mM^–1^ ms^–1^
Dissociation constant for CaMCa4-CaMKII binding	*K*_*CaMKII*_	0.045	μM
CaMKII autophosphorylation rate	$k_{CaMKII}^{Auto}$	10	s^–1^
Total PP1 concentration	[*PP*1]_*T*_	10	μM
Rate constant for PP1 dephosphorylation of pCaMKII	*k*_*PP*1_	1.72	s^–1^

Note that the diffusion constant for *CaMCa*4 was set as *D*_CaM_, and those for *CaMKII_CaMCa*4 and *pCaMKII_CaMCa*4 were set as *D*_CaMKII_.

4Ca+CaM⇋CaMCa4 (24)

The forward and backward reaction rates were specified as $k_{CaM}^{on}$ and $k_{CaM}^{on}\times K_{CaM}$, respectively, with *K*_*CaM*_ defining the dissociation constant related to this binding. Then, the binding of *CaMCa*4 to CaMKII (*CaMKII*) to form a complex (*CaMKII_CaMCa*4*)* complex was modeled as:

CaMCa4+CaMKII⇋CaMKII_CaMCa4 (25)

The forward and backward reaction rates were specified as $k_{CaMKII}^{on}$ and $k_{CaMKII}^{on}\times K_{CaMKII}$, respectively, with *K*_*CaMKII*_ defining the dissociation constant related to this binding. Note that *CaMKII_CaMCa*4 was alternately represented as CaMKII*, with the * representing activation (*e*.*g*., Fig 2*G*). Autophosphorylation of CaMKII was modeled using simple first order kinetics [45,48,169] as:

$$CaMKII\_CaMCa4\overset{}{\to }pCaMKII\_CaMCa4$$ (26)

with the associated reaction rate specified as $k_{CaMKII}^{Auto}$. Note that *CaMKII_CaMCa*4 was alternately represented as pCaMKII*, with the *p* representing phosphorylation (*e*.*g*., Fig 2*G*). Finally, the dephosphorylation of CaMKII [50] occurred through protein phosphatase 1 (*PP*1):

$$pCaMKII\_CaMCa4+PP1\overset{}{\to }CaMKII\_CaMCa4$$ (27)

with the associated reaction rate specified as *k*_*PP*1_. Apart from the radial and longitudinal diffusion of calcium (Eq 16), signaling components represented in Eqs 24–26 also were subjected to radial as well as longitudinal diffusion:

$$\frac{\partial [X]}{\partial t}=D_{X}\nabla^{2}[X]$$ (28)

where *X* represented any of *CaM*, *CaMCa*4, *CaMKII*, *CaMKII_CaMCa*4 or *pCaMKII_CaMCa*4. As initial conditions, *CaM* was set to the total calmodulin concentration, [*CaM*]_T_, *CaMKII* was set to the total CaMKII concentration, [*CaMKII*]_T_, with *CaMCa*4, *CaMKII_CaMCa*4 and *pCaMKII_CaMCa*4 set to zero. The total concentrations and the diffusion constants for these signaling components are listed in Table 2.

### Measurements and data representation

Electrical measurements from the model were recorded employing established procedures [39,55] and are detailed below. To measure the backpropagation of action potentials into dendrites [16,39,147], an action potential was initiated at the soma (2 nA current for 1 ms) and the amplitude of the backpropagating action potential (bAP) was measured at various locations along the somatoapical trunk (Fig 1*C*). Input resistance (*R*_in_) was measured from the local voltage response to a local injection of a 100-pA hyperpolarizing current pulse. The ratio of the steady-state voltage response to the injected current amplitude was taken as the *R*_in_ for that location, and the procedure was repeated for all locations along the somatoapical trunk to construct the *R*_in_ functional map (Fig 1*D*).

To avoid ambiguities with reference to distance representations, distance from the synapse toward the terminal and trunk were designated positive and negative values, respectively (Fig 2*A and* 2*B*). The spatiotemporal spread of each signaling component was represented as a kymograph with the *X*-axis representing time, the *Y*-axis representing space (distance from the synapse towards the trunk and terminal), with the color code representing the numerical value of the component that was plotted. The spread of signaling microdomains through the analyzed signal pathway was depicted as a flow chart of kymographs [8]. The extent of the microdomain was quantified as the area under the curve (AUC) of the plot depicting the maximum value of the signaling component as a function of distance from the synapse (*e*.*g*., Fig 2*F*). In computing the AUC for individual compartments, whereas the entire spatial stretch of the compartment was employed for the spine head and spine neck, the dendritic AUC was computed over the span of 50 μm on either side of the synaptic (or spine) location. When the signaling spread was quantified for different parametric configurations, these plots were computed for each parametric configuration (*e*.*g*., Fig 4*G*) and the AUC values obtained from these plots were assessed as a function of the parameter that was being varied (*e*.*g*., Fig 4*K*). With such quantification, an increase or a decrease in the computed AUC would be a measure of enhancement or suppression, respectively, in the spatial spread of the corresponding signaling microdomain. In performing sensitivity analyses with reference to several critical parameters, the default value associated with each parameter was either increased or decreased two-fold (*e*.*g*., Fig 5) to assess the impact of such a change in the microdomain spread (quantified as the AUC mentioned above).

### Dendritic spine morphology and physiology

For simulations where the synapse was localized on a spine (Figs 9–15), a spine neck (length 1 μm × 0.1 μm diameter) connected to a spine head (length 0.5 μm × 0.5 μm diameter) were added at the center of the oblique dendritic shaft (Fig 9*A*). The spine-head had 10 compartments whereas the spine-neck had 20 compartments, making the size of each compartment to be ~50 nm. The spine had the same passive and active conductances as that of the center of the oblique dendrite from which it originated: *R*_*m*_ = 125 kΩ.cm^2^, *R*_*a*_ = 120 Ω.cm, ${\bar{g}}_{Na}$ = 16 mS/cm^2^, ${\bar{g}}_{KDR}$ = 10 mS/cm^2^, ${\bar{g}}_{KA}$ = 60.55 mS/cm^2^, ${\bar{g}}_{h}$ = 68.75 μS/cm^2^, ${\bar{g}}_{CaT}$ = 285.7 μS/cm^2^. A single synapse containing colocalized AMPA and NMDA receptors was placed at the center of the spine head. The AMPAR permeability was set such that the unitary EPSP amplitude at the soma was ~0.2 mV to match experimental observations [64,170,171], and was set at ${\bar{P}}_{AMPA}$ = 15 nm/s (somatic voltage with spine: 0.22 mV; somatic voltage without spine: 0.25 mV). The NMDAR permeability was set at 1.5× of the AMPAR permeability.

### Spine density analysis

To study the impact of spine density on the spatiotemporal spread of biochemical microdomains, we incorporated several spines throughout the oblique dendrite under consideration. Each of these spines had the same morphology as the synapse-containing spine (Fig 9*A*): spine-neck (length 1 μm × 0.1 μm diameter) connected to a spine-head (length 0.5 μm × 0.5 μm diameter). Each spine had the same passive and active conductances as that of the oblique dendritic compartment from which it originated, including the calcium handling mechanisms. To compare the effect of different densities of spines on the spatiotemporal spread of biochemical microdomains in active dendrites, we compared simulation outcomes in a control (one synapse-containing spine; ~0 spine/μm otherwise) outcomes where the oblique dendrite was populated with spines at four distinct densities. Specifically, the four other cases were built with 100, 200, 500 and 1000 spines (corresponding to ~0.5 spines/μm, ~1 spine/μm, ~2.6 spines/μm and ~5 spines/μm, respectively) distributed randomly (compartments chosen from a uniform distribution) throughout the 2000 compartments of the 193-μm length of the dendritic oblique. All spines except for the synapse-carrying spine (Fig 9*A*) were devoid of any synaptic connections. With this morphological configuration that reflected the characteristics of a hippocampal pyramidal neuron dendrite, we applied TBS through the central synapse-containing spine and looked at the effects of the spatiotemporal kinetics of each species in our chosen biochemical pathway with different spine densities. We tested the effects of *A*-type K^+^ channel and *T*-type Ca^2+^ channel densities for the 1000 spine (density = ~5 spines/μm) case, as this spine density represented the closest approximation to experimental evidence [93].

### Background synaptic activity

For simulating background synaptic activity impinging on the neuron, we incorporated balanced excitation and inhibition so as to keep the average resting membrane potential (RMP) at around –65 mV [172]. One excitatory synapse was placed at each compartment of the somato-apical dendritic arbor within a 300 μm radial distance. Similarly, one inhibitory synapse was placed at each compartment within a radial distance of 50 μm perisomatically, including both apical and basal segments. For both the excitatory and inhibitory synaptic populations, independent random spike generators, each firing at an average rate of 4 Hz was used for input stimulation of each synapse. All the synapses were modeled using an Ohmic formulation with the current through the synapse defined as:

$$i_{syn}\left(t\right)=g_{syn}\left(t\right)\left(V-E_{R}\right)$$ (29)

where *g*_*syn*_ (*t*) defined the time-dependent evolution of each synapse after the onset of an afferent spike, and *E*_R_ defined the reversal potential for the synaptic receptors (*E*_R_ = 0 mV for excitatory synapses and *E*_R_ = –80 mV for inhibitory synapses). *g*_*syn*_ (*t*) was modeled using a double exponential synaptic formulation:

$$g_{syn}(t)=\bar{g}\left[exp\left(-t/\tau_{d}\right)-exp\left(-t/\tau_{r}\right)\right]$$ (30)

where $\bar{g}$ defined the maximal conductance of each synapse set at 0.1 nS for excitatory synapses and 0.6 nS for inhibitory synapses. *τ*_*r*_ (= 2 ms) was the synaptic rise time constant and *τ*_*d*_ (= 10 ms) was the decay time constant for all the synapses. Upon stimulation with such randomized background activity, the mean somatic RMP was found to be –64.33 mV ± 0.74 mV (Fig 10*A*).

### Computer simulations and analysis

All simulations were performed in the *NEURON* simulation environment [144]. The resting membrane potential of the model neuron was fixed at –65 mV. For all experiments, the simulation temperature was set at 34°C and ion channel kinetics were appropriately adjusted according to their experimentally determined *Q*_10_ coefficients. The integration time step was fixed at 25 μs for all simulations to avoid numerical errors in the solution to the differential equations. Data analysis was performed using custom-built software under the IGOR Pro (Wavemetrics Inc., USA) programming environment. The NEURON codes employed to perform the simulations reported in this article are available at the following URL: https://senselab.med.yale.edu/ModelDB/ShowModel.cshtml?model=244848. An updated version of the code in the website fixes and accounts for a volume-scaling bug (See S1 Fig).

All biochemical reactions involving Ca, CaM and CaMKII, their forward rate constants and dissociation constants are listed in S2 Table.

## Supporting information

S1 Text Details of kinetic models of ion channels employed in this study. (PDF)

S1 FigOutcomes of simulations in an updated model that fixes a volume-scaling bug confirm the role active dendritic conductances in regulating signaling spread.(*A*) Fig 2*D* for the updated model, showing maximum value of cytosolic calcium concentration, ${\left[Ca\right]}_{c}^{max}$ plotted as a function of distance from the synapse. (*B*) Fig 2*F* for the updated model, showing maximum value of phosphorylated CaMKII concentration, [*pCaMKII*]^max^ plotted as a function of distance from the synapse. (*C*) *Top*, Fig 4*H* and 4*I* for the updated model, showing the peak value (left) and area under the curve, AUC (right) of ${\left[Ca\right]}_{c}^{max}$. *Bottom*, Fig 4*J* and 4*K* for the updated model, showing the peak value (left) and AUC (right) of [*pCaMKII*]^max^. All graphs are plotted against ${\bar{g}}_{KA}$, depicting the suppression of the spread of calcium and pCamKII microdomains by *A-*type potassium channels. (*D*) *Top*, Fig 6*H* and 6*I* for the updated model, showing the peak value (left) and area under the curve, AUC (right) of ${\left[Ca\right]}_{c}^{max}$. *Bottom*, Fig 6*J* and 6*K* for the updated model, showing the peak value (left) and AUC (right) of [*pCaMKII*]^max^. All graphs are plotted against ${\bar{g}}_{CaT}$, depicting the enhancement of the spread of calcium and pCamKII microdomains by *T-*type calcium channels. All oblique channel parameters are the same as those listed in S1 Table for the specified figures.(TIF)

S1 Table Table containing values of different channel conductances, in the oblique dendrite shown in Fig 2*A*, employed for simulations presented in Figs 1–15. (PDF)

S2 Table Table containing biochemical reactions involving Ca, CaM and CaMKII, their forward rate constants and dissociation constants. (PDF)
