# Supplementary material for: Active dendrites regulate the spatiotemporal spread of signaling microdomains
Source: PLoS Comput Biol. 2018 Nov 1;14(11):e1006485. doi: 10.1371/journal.pcbi.1006485 (PMC6233924; doi:10.1371/journal.pcbi.1006485)
Supplement: S1 Text — (PDF) [file pcbi.1006485.s001.pdf]

Active dendrites regulate the spatiotemporal spread of signaling microdomains

Reshma Basak and Rishikesh Narayanan

Cellular Neurophysiology Laboratory, Molecular Biophysics Unit, Indian Institute of Science, Bangalore, India.

SUPPORTING INFORMATION

S1 Text: Supplementary Methods …………………………………………………… 2

S1 Text: Supplementary Methods

Kinetics of channel models employed

The NaF, KDR, KA and HCN channel models employed the Hodgkin-Huxley formulation [1], whereas the CaT and CaR channels employed the Goldman–Hodgkin–Katz formulation [2,3]. The conductance of all the channels was dependent on gating particle dynamics, which followed first-order kinetics as follows:

$$\frac{dm}{dt} = \frac{m_\infty - m}{\tau_m}$$

where voltage-dependent parameters m_∞ and τ_m respectively defined the steady-state value and the time constant of the state variable that governed the gating particle. A temperature was set at 34 °C for all the equations below.

The fast sodium channel

The NaF channel model was adopted from [4-7].

Current:

$$I_{NaF} = \bar{g}_{NaF} m^3 h s (V - E_{Na})$$

Where $E_{Na} = +55$ mV

Activation kinetics:

$$m_\infty^{NaF} = \frac{\alpha_m^{NaF}}{\alpha_m^{NaF} + \beta_m^{NaF}} \qquad \tau_m^{NaF} = \frac{0.5}{\alpha_m^{NaF} + \beta_m^{NaF}}$$

$$\alpha_m^{NaF} = \frac{0.4(V+30)}{1-\exp\left(\frac{-(V+30)}{7.2}\right)} \qquad \beta_m^{NaF} = \frac{0.124(V+30)}{\exp\left(\frac{(V+30)}{7.2}\right)-1}$$

if $\tau_m^{NaF} < 0.02$, then $\tau_m^{NaF} = 0.02$ ms

Fast inactivation kinetics:

$$h_\infty^{NaF} = \frac{1}{1+\exp\left(\frac{V+50}{4}\right)} \qquad \tau_h^{NaF} = \frac{0.5}{\alpha_h^{NaF} + \beta_h^{NaF}}$$

$$\alpha_h^{NaF} = \frac{0.03(V+45)}{1-\exp\left(\frac{-(V+45)}{1.5}\right)} \qquad \beta_h^{NaF} = \frac{0.01(V+45)}{\exp\left(\frac{(V+45)}{1.5}\right)-1}$$

if $\tau_h^{NaF} < 0.5$, then $\tau_h^{NaF} = 0.5$ ms

Slow inactivation kinetics:

$$s_\infty^{NaF} = \frac{1+b_s\exp((V+58)/2)}{1+\exp((V+58)/2)} \qquad \tau_s^{NaF} = \frac{3\times10^4\beta_s^{NaF}}{1+\alpha_s^{NaF}}$$

$$\alpha_s^{NaF} = \exp(0.45(V+60)) \qquad \beta_i^{NaF} = \exp(0.09(V+60))$$

if $\tau_i^{NaF} < 10$, then $\tau_s^{NaF} = 10$ ms

$b_s = 0.8$ at apical dendrites
$b_s = 1$ elsewhere

The delayed rectifier potassium channel

The KDR channel model was adopted from [4,6].

Current:

$$I_{KDR} = \bar{g}_{KDR}n(V-E_k)$$

Where $E_k = -90$ mV

Activation kinetics:

$$n_\infty^{KDR} = \frac{1}{1+\alpha_n^{KDR}} \qquad \tau_n^{KDR} = \frac{50\beta_n^{KDR}}{1+\alpha_n^{KDR}}$$

$$\alpha_n^{KDR} = \exp(-0.11(V-13)) \qquad \beta_n^{KDR} = \exp(-0.08(V-13))$$

if $\tau_n^{KDR} < 2$, then $\tau_n^{KDR} = 2$ ms

The A-type potassium channel

The KA channel model was adopted from [4,6]. Current through the proximal KA channel:

$$I_{KA_P} = \bar{g}_{KA_P} nl(V - E_k)$$

Activation kinetics:

$$n_\infty^{KA_P} = \frac{1}{1+\alpha_n^{KA_P}} \qquad \tau_n^{KA_P} = \frac{4\beta_n^{KA_P}}{1+\alpha_n^{KA_P}}$$

$$\alpha_n^{KA_P} = \exp\left(-0.038\left(1.5 + \frac{1}{1+\exp(V+40)/5}\right)(V-11)\right)$$

$$\beta_n^{KA_P} = \exp\left(-0.038\left(0.825 + \frac{1}{1+\exp(V+40)/5}\right)(V-11)\right)$$

if $\tau_n^{KA_P} < 0.1$, then $\tau_n^{KA_P} = 0.1$ ms

Inactivation kinetics:

$$l_\infty^{KA_P} = \frac{1}{1+\alpha_l^{KA_P}} \qquad \tau_l^{KA_P} = 0.26(V+50)$$

$$\alpha_l^{KA_P} = \exp(0.11(V+56))$$

if $\tau_l^{KA_P} < 2$, then $\tau_l^{KA_P} = 2$ ms

Current through the distal KA channel:

$$I_{KA_D} = \bar{g}_{KA_D} nl(V - E_k)$$

Activation kinetics:

$$n_\infty^{KA_D} = \frac{1}{1+\alpha_n^{KA_D}} \qquad \tau_n^{KA_D} = \frac{2\beta_n^{KA_D}}{1+\alpha_n^{KA_D}}$$

$$\alpha_n^{KA_D} = \exp\left(-0.038\left(1.8 + \frac{1}{1+\exp(V+40)/5}\right)(V+1)\right)$$

$$\beta_n^{KA_D} = \exp\left(-0.038\left(0.7 + \frac{1}{1+\exp(V+40)/5}\right)(V+1)\right)$$

if $\tau_n^{KA_D} < 0.1$, then $\tau_n^{KA_D} = 0.1\,\mathrm{ms}$

Inactivation kinetics:

$$l_\infty^{KA_D} = \frac{1}{1+\alpha_l^{KA_D}} \qquad \tau_l^{KA_D} = 0.26(V+50)$$

$$\alpha_l^{KA_D} = \exp(0.11(V+56))$$

if $\tau_l^{KA_D} < 2$, then $\tau_l^{KA_D} = 2\,\mathrm{ms}$

The hyperpolarization-activation cyclic-nucleotide-gated non-specific cationic channel

The HCN channel model was adopted from [8].

Current:

$$I_{HCN} = \bar{g}_{HCN} l (V - E_{HCN})$$

Where $E_{HCN} = -30$ mV

Activation kinetics:

$$l_\infty^{HCN} = \frac{1}{1+\exp\left(\frac{V+75}{8}\right)} \qquad \tau_l^{HCN} = \frac{78.21\beta_l^{HCN}}{1+\alpha_l^{HCN}}$$

$$\alpha_l^{HCN} = \exp(0.083(V+75)) \qquad \beta_l^{HCN} = \exp(0.033(V+75))$$

The T-type calcium channel

The CaT channel model was adopted from [5,9,10]:

Current:

$$I_{CaT} = \bar{g}_{CaT} m^2 h GHK_{Ca}(V, [Ca]_i, [Ca]_o)$$

where,

$$GHK_{Ca} = \left(\frac{1 - \frac{[Ca]_i}{[Ca]_o} \exp\left(\frac{2vF}{RT}\right)}{1 - \exp\left(\frac{2vF}{RT}\right)} \right)$$

Activation kinetics:

$$m_\infty^{CaT} = \frac{1}{1 + \exp\left(\frac{-(V+60)}{10}\right)} \qquad \tau_m^{CaT} = \frac{5.87\beta_m^{CaT}}{1 + \alpha_m^{CaT}}$$

$$\alpha_m = \exp(0.076(V+28)) \qquad \beta_m = \exp(0.046(V+28))$$

$$\text{if } \tau_m^{CaT} < 0.2\text{, then } \tau_m^{CaT} = 0.2 \text{ ms}$$

Inactivation kinetics:

$$h_\infty^{CaT} = \frac{1}{1 + \exp\left(\frac{(V+85)}{10}\right)} \qquad \tau_h^{CaT} = \frac{66.67\beta_m^{CaT}}{1 + \alpha_m^{CaT}}$$

$$\alpha_h = \exp(0.132(V+75)) \qquad \beta_h = \exp(0.079(V+75))$$

$$\text{if } \tau_h^{CaT} < 10\text{, then } \tau_h^{CaT} = 10 \text{ ms}$$

The R-type calcium channel

The CaR channel model was adopted from [5,9,10].

Current:

$$I_{CaR} = \bar{g}_{CaR} m^3 h GHK_{Ca}(V, [Ca]_i, [Ca]_o)$$

Activation kinetics:

$$m_\infty^{CaR} = \frac{1}{1 + \exp\left(\frac{-(V-3)}{8.3}\right)} \qquad \tau_m^{CaR} = 0.246$$

Inactivation kinetics:

$$h_\infty^{CaR} = \frac{1}{1 + \exp\left(\frac{(V+39)}{9.2}\right)} \qquad \tau_h^{CaR} = 14.18$$

References

1. Hodgkin AL, Huxley AF (1952) A quantitative description of membrane current and its application to conduction and excitation in nerve. J Physiol 117: 500-544.
2. Goldman DE (1943) Potential, Impedance, and Rectification in Membranes. J Gen Physiol 27: 37-60.
3. Hodgkin AL, Katz B (1949) The effect of sodium ions on the electrical activity of giant axon of the squid. J Physiol 108: 37-77.
4. Migliore M, Hoffman DA, Magee JC, Johnston D (1999) Role of an A-type K+ conductance in the back-propagation of action potentials in the dendrites of hippocampal pyramidal neurons. J Comput Neurosci 7: 5-15.
5. Magee JC, Johnston D (1995) Characterization of single voltage-gated Na+ and Ca2+ channels in apical dendrites of rat CA1 pyramidal neurons. J Physiol 487 (Pt 1): 67-90.
6. Hoffman DA, Magee JC, Colbert CM, Johnston D (1997) K+ channel regulation of signal propagation in dendrites of hippocampal pyramidal neurons. Nature 387: 869-875.
7. Colbert CM, Magee JC, Hoffman DA, Johnston D (1997) Slow recovery from inactivation of Na+ channels underlies the activity-dependent attenuation of dendritic action potentials in hippocampal CA1 pyramidal neurons. J Neurosci 17: 6512-6521.
8. Magee JC (1998) Dendritic hyperpolarization-activated currents modify the integrative properties of hippocampal CA1 pyramidal neurons. J Neurosci 18: 7613-7624.
9. Johnston D, Magee JC, Colbert CM, Cristie BR (1996) Active properties of neuronal dendrites. Annu Rev Neurosci 19: 165-186.
10. Shah MM, Migliore M, Valencia I, Cooper EC, Brown DA (2008) Functional significance of axonal Kv7 channels in hippocampal pyramidal neurons. Proc Natl Acad Sci U S A 105: 7869-7874.
