# Supplementary figures and images for: Active dendrites regulate the spatiotemporal spread of signaling microdomains
Source: PLoS Comput Biol. 2018 Nov 1;14(11):e1006485. doi: 10.1371/journal.pcbi.1006485 (PMC6233924; doi:10.1371/journal.pcbi.1006485)

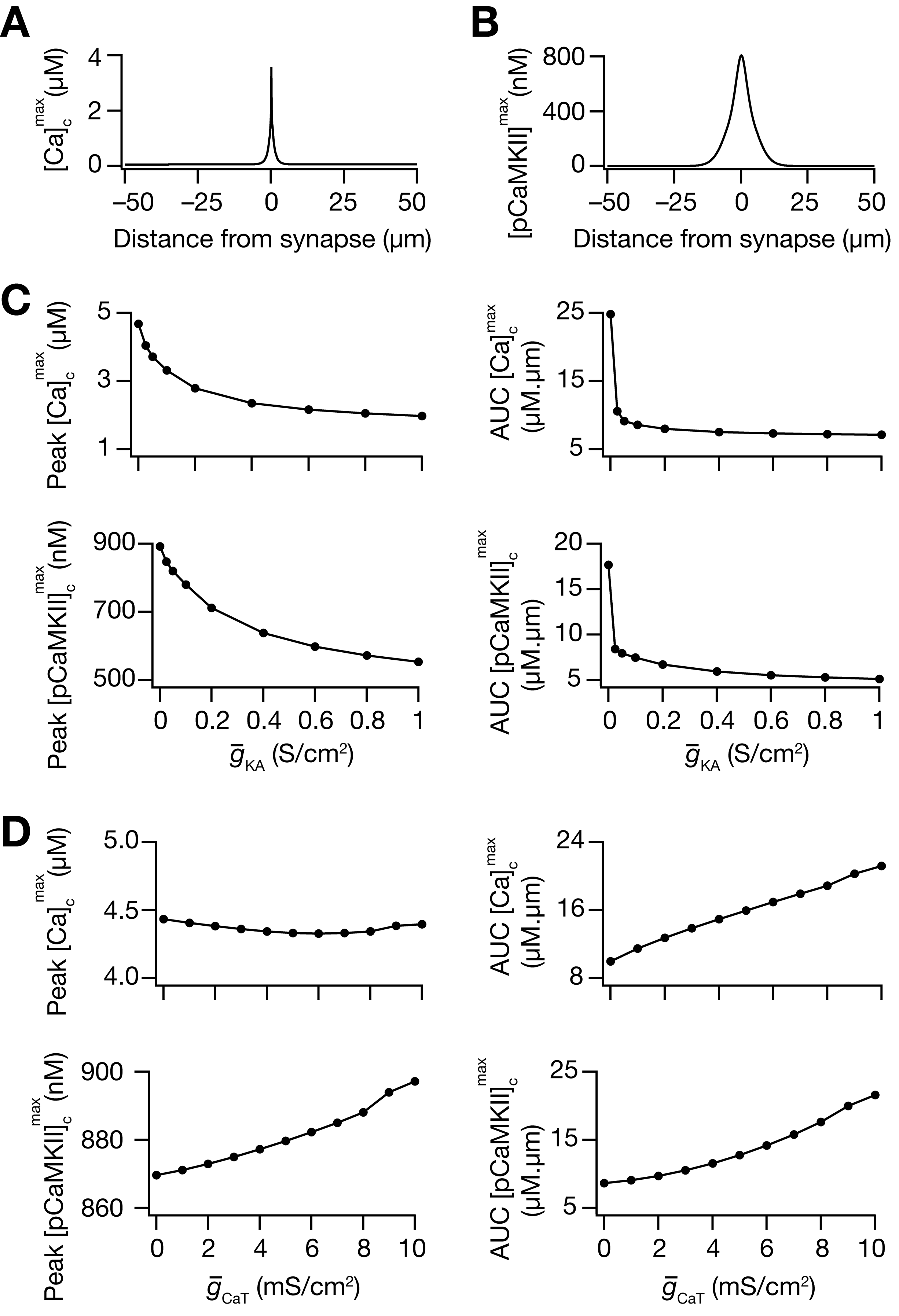

Supplement: S1 Fig — (A) Fig 2D for the updated model, showing maximum value of cytosolic calcium concentration, [Ca]cmax plotted as a function of distance from the synapse. (B) Fig 2F for the updated model, showing maximum value of phosphorylated CaMKII concentration, [pCaMKII]max plotted as a function of distance from the synapse. (C) Top, Fig 4H and 4I for the updated model, showing the peak value (left) and area under the curve, AUC (right) of [Ca]cmax. Bottom, Fig 4J and 4K for the updated model, showing the peak value (left) and AUC (right) of [pCaMKII]max. All graphs are plotted against g¯KA, depicting the suppression of the spread of calcium and pCamKII microdomains by A-type potassium channels. (D) Top, Fig 6H and 6I for the updated model, showing the peak value (left) and area under the curve, AUC (right) of [Ca]cmax. Bottom, Fig 6J and 6K for the updated model, showing the peak value (left) and AUC (right) of [pCaMKII]max. All graphs are plotted against g¯CaT, depicting the enhancement of the spread of calcium and pCamKII microdomains by T-type calcium channels. All oblique channel parameters are the same as those listed in S1 Table for the specified figures. (TIF) [file pcbi.1006485.s002.tif]
