# Supplementary material for: Active dendrites regulate the spatiotemporal spread of signaling microdomains
Source: PLoS Comput Biol. 2018 Nov 1;14(11):e1006485. doi: 10.1371/journal.pcbi.1006485 (PMC6233924; doi:10.1371/journal.pcbi.1006485)
Supplement: S1 Table — (PDF) [file pcbi.1006485.s003.pdf]

S1 Table

Table containing parametric values used in each figure at the trunk-end (–Trunk), synaptic location (–Syn; highlighted in grey) and the terminal-end (–Term) of the oblique highlighted in Figure 2*A*. The values provided for Figure 1 constitute baseline values.

Parameter (Unit) \ Figure No.	1	2	3	4	5	6	7	8	9	10	11	12	13	14	15 (A&C)	15 (B&D)
R_m–whole ob (kΩ/cm^2)	125	125	125	125	125	125	125	125	125	125	125	125	125	125	125	125
R_a–whole ob (Ω.cm)	120	120	120	120	120	120	120	120	120	120	120	120	120	120	120	120
$\bar{g}_{Na}$ –Trunk (mS/cm^2)	16	16	16	16	16	16	16	16	16	16	16	16	16	16	16	16
$\bar{g}_{Na}$ –Syn (mS/cm^2)	16	16	16	16	16	16	16	16	16	16	16	16	16	16	16	16
$\bar{g}_{Na}$ –Term (mS/cm^2)	16	16	16	16	16	16	16	16	16	16	16	16	16	16	16	16
$\bar{g}_{KDR}$ –Trunk (mS/cm^2)	10	10	10	10	10	10	10	10	10	10	10	10	10	10	10	10
$\bar{g}_{KDR}$ –Syn (mS/cm^2)	10	10	10	10	10	10	10	10	10	10	10	10	10	10	10	10
$\bar{g}_{KDR}$ –Term (mS/cm^2)	10	10	10	10	10	10	10	10	10	10	10	10	10	10	10	10
$\bar{g}_{KA}$ –Trunk (mS/cm^2)	51.75	51.75	51.75	0 – 1000	0 – 1000	0	0	0 – 1000	51.75	51.75	0 – 1000	51.75	51.75	51.75	0 – 1000	51.75
$\bar{g}_{KA}$ –Syn (mS/cm^2)	60.55	60.55	60.55	0 – 1000	0 – 1000	0	0	0 – 1000	60.55	60.55	0 – 1000	60.55	60.55	60.55	0 – 1000	60.55
$\bar{g}_{KA}$ –Term (mS/cm^2)	67.73	67.73	67.73	0 – 1000	0 – 1000	0	0	0 – 1000	67.73	67.73	0 – 1000	67.73	67.73	67.73	0 – 1000	67.73
$\bar{g}_{CaT}$ –Trunk (mS/cm^2)	0.19	0.19	0.19	0	0	0 – 10	0 – 10	0 – 10	0.19	0.19	0.19	0 – 10	0.19	0.19	0.19	0 – 10
$\bar{g}_{CaT}$ –Syn (mS/cm^2)	0.29	0.29	0.29	0	0	0 – 10	0 – 10	0 – 10	0.29	0.29	0.29	0 – 10	0.29	0.29	0.29	0– 10
$\bar{g}_{CaT}$ –Term (mS/cm^2)	0.42	0.42	0.42	0	0	0 – 10	0 – 10	0 – 10	0.42	0.42	0.42	0 – 10	0.42	0.42	0.42	0 – 10
$\bar{g}_{h}$ –Trunk (µS/cm^2)	48.26	48.26	48.26	0	0	0	0	0	48.26	48.26	48.26	48.26	48.26	48.26	48.26	48.26
$\bar{g}_{h}$ –Syn (µS/cm^2)	68.77	68.77	68.77	0	0	0	0	0	68.77	68.77	68.77	68.77	68.77	68.77	68.77	68.77
$\bar{g}_{h}$ –Term (µS/cm^2)	95.10	95.10	95.10	0	0	0	0	0	95.10	95.10	95.10	95.10	95.10	95.10	95.10	95.10
$\bar{g}_{CaR}$ –Trunk (mS/cm^2)	0	0	0	100	100	0	0	0,100	0	0	0	0	0	0	0	0
$\bar{g}_{CaR}$ –Syn (mS/cm^2)	0	0	0	100	100	0	0	0,100	0	0	0	0	0	0	0	0
$\bar{g}_{CaR}$ –Term (mS/cm^2)	0	0	0	100	100	0	0	0,100	0	0	0	0	0	0	0	0
