# Supplementary material for: Active dendrites regulate the spatiotemporal spread of signaling microdomains
Source: PLoS Comput Biol. 2018 Nov 1;14(11):e1006485. doi: 10.1371/journal.pcbi.1006485 (PMC6233924; doi:10.1371/journal.pcbi.1006485)
Supplement: S2 Table — (PDF) [file pcbi.1006485.s004.pdf]

S2 Table

Table containing detailed biochemical reactions involving Ca, CaM and CaMKII, their forward rate constants and dissociation constants.

**Reaction**	**Forward reaction rate ($mM^{-1}\ ms^{-1}$)**	**Dissociation constant (µM)**
$CaM + Ca \rightleftharpoons CaMCa$	8.4848	1.0001
$CaMCa + Ca \rightleftharpoons CaMCa2$	8.4848	1.0001
$CaMCa2 + Ca \rightleftharpoons CaMCa3$	8.4848	1.0001
$CaMCa3 + Ca \rightleftharpoons CaMCa4$	8.4848	1.0001
$CaM + CaMKII \rightleftharpoons CaMKII\_CaM$	0.2	13500
$CaMCa + CaMKII \rightleftharpoons CaMKII\_CaMCa$	0.8	3375.0084
$CaMCa2 + CaMKII \rightleftharpoons CaMKII\_CaMCa2$	2	1125
$CaMCa3 + CaMKII \rightleftharpoons CaMKII\_CaMCa3$	100.004	0.225
$CaMCa4 + CaMKII \rightleftharpoons CaMKII\_CaMCa4$	100.004	0.045
$CaMKII\_CaM + Ca \rightleftharpoons CaMKII\_CaMCa$	4	5
$CaMKII\_CaMCa + Ca \rightleftharpoons CaMKII\_CaMCa2$	100.004	0.2
$CaMKII\_CaMCa2 + Ca \rightleftharpoons CaMKII\_CaMCa3$	100.004	0.02
$CaMKII\_CaMCa3 + Ca \rightleftharpoons CaMKII\_CaMCa4$	100.004	1
